# Voltage-gated sodium channels in the nervous system: Molecular physiology to therapeutic interventions

**DOI:** 10.4103/NRR.NRR-D-25-00260

**Published:** 2025-08-13

**Authors:** Ni Li, Lin Yan, Anna Peng, Xuefei Fu, Huan Qin, Kai Yao

**Affiliations:** 1 Institute of Visual Neuroscience and Stem Cell Engineering, Wuhan University of Science and Technology, Wuhan, Hubei Province, China; 2 College of Life Sciences and Health, Wuhan University of Science and Technology, Wuhan, Hubei Province, China

**Keywords:** autism spectrum disorder, channelopathies, clinical trial, Dravet syndrome, epilepsy, familial hemiplegic migraine, gene therapy, nerve regeneration, neuronal regeneration, renin-angiotensin system, retinal degeneration

## Abstract

Voltage-gated sodium channels are essential ionic-conductance pathways in the nervous system, which play an irreplaceable role in modulating neuronal excitability and signal transduction. This review comprehensively analyzes the molecular mechanisms and pathophysiological significance of voltage-gated sodium channels, with particular emphasis on elucidating the molecular-action mechanisms of the distinct subtypes of these channels, including Nav1.1, Nav1.2, and Nav1.6, across various neurological disorders such as familial hemiplegic migraine, epilepsy, autism spectrum disorder, and retinal dysfunction. This review also provides a comprehensive overview of the pathogenic mechanisms associated with voltage-gated sodium channels, and systematically clarifies the evolutionary pathway of treatment strategies from conventional to innovative approaches. It analyzes two major categories of conventional sodium channel blockers and their applications: antiepileptic drugs (such as carbamazepine, lamotrigine, and phenytoin) and antiarrhythmic drugs (such as lidocaine, flecainide, and quinidine). However, these conventional blockers show limitations because of the lack of selectivity, driving research toward more precise therapeutic directions. Additionally, this review evaluates gabapentin, cannabidiol, and calcium channel blockers with different mechanisms of action. These drugs modulate neuronal excitability from multiple perspectives, providing diverse options for symptom relief. This review also highlights advances in gene therapy for specific diseases, such as STK-001, which promotes effective splicing of the sodium channel voltage-gated type 1 alpha subunit (*SCN1A*) gene, and ETX101, which utilizes adeno-associated virus 9 vectors to deliver engineered transcription factors. These two agents provide targeted therapeutic solutions for Dravet syndrome. Furthermore, this review summarizes some innovative therapeutic agents in clinical trials, including PRAX-222 (for *SCN2A* gain-of-function mutation-related epilepsy), which has received Food and Drug Administration orphan drug designation, and the selective Nav1.6 inhibitor NBI-921352 (for *SCN8A*-related epilepsy). Collectively, this review comprehensively compares the advantages and disadvantages of conventional drugs and gene therapy and envisions future treatment strategies that integrate the strengths of both approaches, facilitating personalized precision medicine to provide more accurate and effective treatment options for patients with ion channel diseases.

## Introduction

Neurological diseases are complex and wide-ranging disorders that can damage the structure and function of the nervous system (Peng et al., 2025; Yang et al., 2025). The nervous system consists of central and peripheral nervous systems, which together form a network responsible for information processing and regulation (Alborghetti et al., 2025). This network regulates various functions of the human body using neurotransmitters and electrical signals (Carvajal Alegria et al., 2016). The pathogenic mechanisms underlying neurological diseases are highly complex and involve multiple contributing factors. Neurological diseases can arise from gene mutations that lead to abnormal protein expression or functional changes. Additionally, certain environmental factors may lead to the development of these neurological diseases by triggering oxidative stress, causing mitochondrial dysfunction, or provoking neuroinflammatory responses. Common neurological diseases, such as migraine (Aguilar-Shea et al., 2022), autism spectrum disorder (ASD) (Wang et al., 2023), and epilepsy (Neri et al., 2022), typically involve the combined action of multiple pathological pathways. Although modern medicine has made substantial strides in controlling symptoms and slowing disease progression, completely curing these diseases remains a formidable challenge. Voltage-gated sodium channels (VGSCs) play a central role in regulating the functions of the nervous system. Nine different subtypes of VGSCs have been identified, ranging from Nav1.1 to Nav1.9. Each subtype is encoded by specific genes, namely the *SCN1–5* and *SCN7–10* genes (Ahern et al., 2016). In the central and peripheral nervous systems of mammals, these ion channels exhibit highly specific expression patterns.

VGSCs can precisely regulate the changes in membrane potential and the transmembrane transport of sodium ions. This is crucial for the generation and conduction of neuronal action potentials. This complex regulatory mechanism maintains the appropriate excitability of the nervous system. Abnormal functioning of VGSCs may result in neuronal excitability disorder, triggering a series of diseases. Such diseases include familial hemiplegic migraine (FHM) (Villar-Martinez et al., 2024), epilepsy (Dong et al., 2024), ASD (Antshel and Russo, 2019), and retinal dysfunction. Abnormal functions of VGSCs of different subtypes often lead to specific clinical manifestations. An in-depth understanding of the structural and functional relationships of VGSCs can provide the basis for formulating targeted therapeutic strategies.

The important roles of VGSCs in neuronal plasticity and regeneration processes have been investigated. During post-injury neural repair, VGSC expression patterns are dynamically modulated, and this process is closely associated with axonal regeneration, synaptic reconstruction, and functional recovery (Hingorani et al., 2024). This review aims to systematically elucidate the core mechanisms of VGSCs in the pathophysiology of various neurological disorders, and to explore their pathogenic mechanisms at the molecular level in depth. Specifically, this review investigates the unique regulatory role of VGSCs as key molecular switches in neuronal plasticity and neural regeneration, providing new perspectives for understanding the intrinsic mechanisms of neurological functional recovery. Through a comprehensive summary of conventional and innovative therapeutic strategies for sodium channelopathies, this review details the evolutionary journey from conventional sodium channel blockers (SCBs) to cutting-edge gene therapies. Furthermore, this review highlights the breakthrough of precision targeting strategies for specific VGSC subtypes in clinical applications, aiming to establish a solid theoretical framework for personalized precision medicine in neurological disorders, and to provide an essential scientific basis for developing safer and more effective novel therapeutic approaches.

## Search Strategy

The articles that address VGSC-related research and were published from 2000–2025 were retrieved through an electronic search of PubMed using the following criteria: voltage-gated sodium channel [MeSH term] AND (disease [MeSH term] OR neurodevelopment/neuroregeneration [MeSH term] OR retinal degeneration [MeSH term] OR treatment [MeSH term]. Additionally, the literature on VGSC disease treatment published before May 2025 was also searched in the PubMed database via the following search terms: voltage-gated sodium channel, autism spectrum disorder, epilepsy, and familial hemiplegic migraine.

## Structure and Characteristics of Voltage-Gated Sodium Channels

VGSCs are complex transmembrane proteins. They are crucial for the functions of nerve cells, muscle fibers, and other excitable cells. During depolarization of the cell membrane, these channels play a core role in the generation of action potentials (Wisedchaisri et al., 2019). The function of the channel depends on its structure and regulatory mechanism. When cells are in a resting state, sodium channels remain closed. At this time, potassium channels are selectively open, and the sodium-potassium pump utilizes ATP to transport ions across the membrane, helping the cell membrane maintain the resting voltage. When the local membrane voltage reaches the threshold due to stimulation, the sodium channel will change its configuration. The activation gate opens, allowing extracellular sodium ions to flow rapidly inward along the concentration gradient. This will lead to rapid depolarization of the membrane, and the voltage rises rapidly to the peak. Subsequently, the inactivation gate of the channel closes to prevent further inflow of sodium ions. This crucial step creates conditions for the membrane voltage to return to the resting state (Chen et al., 2024).

**Figure 1:**
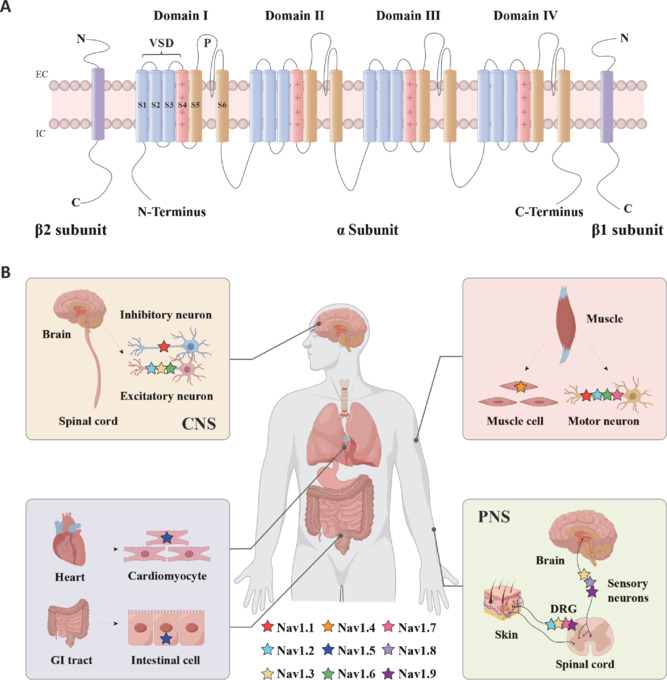
Structure and distribution of voltage-gated sodium channels. (A) Topology of the eukaryotic Nav channel. The channel consists of a central α subunit, which includes four homologous domains (domains I–IV), each containing six transmembrane segments. The fourth transmembrane segment (pink) acts as a voltage sensor because of its positive charge. The loops between the fifth and sixth transmembrane segments (orange) form the ion-selective pore. The β subunits (β1 and β2) are auxiliary subunits that modulate channel function and localization. (B) The distribution of different Nav channel subtypes in the human body. Nav channels demonstrate tissue-specific expression, with distinct isoforms (Nav1.1–Nav1.9) found in various systems. CNS: Central nervous system; DRG: dorsal root ganglion; EC: extracellular; GI: gastrointestinal; IC: intracellular; PNS: peripheral nervous system; VSD: voltage-sensing domain.

The molecular structure of VGSCs is highly repetitive. **Figure 1A** shows that these channels are mainly composed of two transmembrane subunits. The α subunit itself can form an ion channel, while the auxiliary β subunit is assembled together with the α subunit to form a trimer structure. The human body contains nine different alpha subunits, namely Nav1.1 to Nav1.9, and five different β subunits, including β1–β4 and the splicing variant β1B (Alsaloum et al., 2025).

The α subunit is the core part of the channel. It consists of four structurally similar repetitive domains (I–IV), of which each domain contains six transmembrane segments (S1–S6), constituting the basic functional units of channel proteins. In each repetitive structure, the S1–S4 segments jointly form the voltage-sensing domain (Antonaci et al., 2021). Among them, S4, as a key voltage sensor, is capable of responding rapidly to changes in membrane potential (Sanders et al., 2018; Wisedchaisri et al., 2019). The ion-selective filter is located in the central region of the α subunit. It is jointly constructed by the S5 and S6 fragments and the P ring between them, ensuring highly selective passage of sodium ions (Shen et al., 2017). The four α-helical bundles (S1–S4) of the voltage sensor are connected to the central channel structure through the S4–S5 connection peptide, thereby transmitting the voltage signal to the gating system (Payandeh et al., 2011).

The helper β subunits (β1–β4) have three main working parts. The first part is the outer V-type antibody-like (Ig) region that helps cells stick together and proteins interact. The second part is the single spiral segment that crosses the cell membrane, anchoring the protein in place. The third part is the inside tail end that helps break down proteins within the membrane and sends signals to the cell nucleus. The β1B variant retains the outer antibody-like region but lacks the membrane-crossing and internal components. The missing parts are replaced by a different type of tail structure (Williams et al., 2025).

The activation of VGSC stems occurs through the synergistic effects of the α and β subunits (Xu et al., 2019). When the membrane is depolarized, the gating charges (mainly arginine residues) on the S4 fragment of the α subunit undergo voltage-driven displacement first. This conformational change is delivered to the gating system of the channel through the S4–S5 linker peptide, eventually leading to the opening of the pore domain formed by the S5 and S6 fragments and the P-loop. Meanwhile, the β subunit regulates the conformational stability of the voltage sensor through its interaction with the α subunit. The β1/β3 subunits are connected to the α subunit through non-covalent bonds, while the β2/β4 subunits are linked to the α subunit through disulfide bonds. β1 and β3 often accelerate channel inactivation, while β2 has a greater impact on sodium channel membrane expression. This precise coordination ensures accurate control of sodium ion influx, which is crucial for signal transduction in neurons and muscle cells (Mojumder et al., 2007; Wisedchaisri et al., 2019; Chen et al., 2024).

VGSCs are ubiquitous throughout the organism. Their expression levels are particularly high in the central neurons, peripheral neurons, and cardiomyocytes. They play a crucial role in nerve impulse conduction and cardiac electrophysiological activities. **Additional Table 1** systematically summarizes the coding genes, electrophysiological characteristics, and specific regulators of various sodium channel subtypes. **Figure 1B** shows the distribution of the main sodium channel subtypes in body tissues. In the central nervous system, Nav1.1, Nav1.2, Nav1.3, and Nav1.6 are the main subtypes (Martin et al., 2007; Thouta et al., 2022). Meanwhile, Nav1.7, Nav1.8, and Nav1.9 occupy dominant positions in the peripheral nervous system (Catterall, 2012). These subtypes can be functionally classified on the basis of their sensitivity to tetrodotoxin. Tetrodotoxin-sensitive sodium channels are mainly involved in the rapid initial depolarization of the action potential (Lucas et al., 2014; Carrasco et al., 2017; Mattei, 2018; Martinez-Espinosa et al., 2021; Nair et al., 2023). Tetrodotoxin-resistant channels, due to their relatively slow activation and inactivation kinetics, are helpful for the continuous discharge mode (Carrasco et al., 2017; Xiao et al., 2022; Nascimento de Lima et al., 2024).

**Additional Table 1:** Molecular and pharmacological characteristics of voltage-gated sodium channel subtypes

Ion channel subtype	Gene	Primary expression site	Main expression period	Sensitivity to tetrodotoxin	Specific activator/inhibitor	Reference
Nav1.1	*SCN1A*	CNS	Neonatal, infant, and early childhood periods	Sensitive	Hm1a (activator); Hm1b (activator); AA43279 (activator)	Frederiksen et al., 2017; Chow et al., 2020
Nav1.2	*SCN2A*	CNS	Expression is low in the adult brain during fetal and infant stages	Sensitive	μ-Conotoxin KIIIA (inhibitor)	Pan et al., 2019
Nav1.3	*SCN3A*	CNS & PNS	Expression from embryos to adulthood	Sensitive	ICA-121431 (inhibitor)	Réthoré et al., 2022
Nav1.4	*SCN4A*	Skeletal muscle cells	High expression starts late in fetal stage and peaks after birth	Sensitive	μ-Conotoxin GIIIA (inhibitor)	Han et al., 2016
Nav1.5	*SCN5A*	Cardiac muscle cells	Expression is high during embryonic stage, mainly for heart function after birth	Insensitive	Jingzhaotoxin-III (inhibitor)	Rong et al., 2011
Nav1.6	*SCN8A*	CNS & PNS	Low expression in neonates and widespread expression in adults	Sensitive	NBI-921352 (inhibitor) Prax330/GS967 (inhibitor) Centruroides noxius toxin 2 (activator)	Israel et al., 2019; Johnson et al., 2022; Alsaloum et al., 2025
Nav1.7	*SCN9A*	PNS	Expression begins late in embryo and gradually increases after birth	Sensitive	PTx2-3127/PTx23258 (inhibitor) μ-SLPTX-Ssm6a (inhibitor) XEN907 (inhibitor) PF-05089771 (inhibitor)	Chowdhury et al., 2011; Yang et al., 2013; Nguyen et al., 2022; Jakusova et al., 2025
Nav1.8	*SCN10A*	PNS	Expression is low in embryo and peaks in adulthood	Insensitive	VX-150/VX-548 (inhibitor) LTGO-33 (inhibitor) MSD199 (inhibitor) A-803467 (inhibitor) PF-06305591 (inhibitor)	Jarvis et al., 2007; Brown et al., 2019; Gilchrist et al., 2024; Vaelli et al., 2024; McDevitt et al., 2025
Nav1.9	*SCN11A*	PNS	Expression increases after birth and is stable in adulthood	Insensitive	HpTx1 (activator)	Zhou et al., 2020

This table comprehensively summarizes the molecular structural characteristics and pharmacological properties of various voltage-gated sodium channel subtypes. The detailed data on specific activators/inhibitors targeting different sodium channel subtypes provide researchers with important tools for precise identification and targeted intervention. This information provides a solid foundation for studying the pathological mechanisms of sodium channels, offering a reliable theoretical basis and experimental guidance for further exploration of the pathogenesis, clinical features, and potential therapeutic strategies for diseases related to each subtype. CNS: Central nervous system; PNS: peripheral nervous system.

In the nervous system, different subtypes of sodium ion channels exhibit various functional specificities. For instance, the Nav1.1 channel can precisely regulate the discharge threshold of the neuronal action potential, thereby achieving regulation and control of neuronal excitability. These channels also play crucial roles in regulating synaptic transmission (Yamagata et al., 2023). Dysfunction of Nav1.1 is closely related to many central nervous system diseases, including migraine (Shen et al., 2017), epilepsy (Ji et al., 2025), Dravet syndrome (DS; Samanta, 2025), and autism (Osteen et al., 2016).

Nav1.2 shows a relatively high expression level in the excitatory neurons of the central nervous system (Wang et al., 2021). During the early development of mammals, Nav1.2 is the only sodium channel subtype. It is expressed in axons and the initial segments of axons and processes and independently assumes the responsibility for initiation and propagation of action potentials (Spratt et al., 2019). Mutations in the gene expressing Nav1.2 are associated with many neurological disorders, including infantile epilepsy, idiopathic epilepsy, ASD, and intellectual developmental delay (Yamagata et al., 2017; Kruth et al., 2020; Thompson et al., 2023).

During the development of the human brain, the expression of Nav1.3 shows an obvious temporal pattern. The fetal period is the peak period for the expression of this sodium channel. As the brain continues to develop, the expression level of Nav1.3 gradually decreases (Cheah et al., 2013). Nav1.3 has two notable features: it can quickly recover from an inactivated state and simultaneously shows slow inactivation kinetics. These characteristics endow neurons expressing Nav1.3 with low-threshold and high-frequency discharge capabilities. As a result, neurons become more easily excited, and the sensory threshold decreases accordingly (Liao et al., 2023). Nav1.3 channel dysfunction shows a very prominent association with various neurological diseases, including epilepsy (Estacion et al., 2010) and Parkinson disease (Wang et al., 2019).

The action potential generated by Nav1.4 can trigger the release of Ca^2+^ and also cause the contraction response of the entire muscle fiber (Wu et al., 2016). Mutations in Nav1.4 are pathologically associated with many neuromuscular diseases, including hyperkalemic periodic paralysis (Zhao et al., 2024), hypokalemic periodic paralysis type 2 (Maggi et al., 2020), paramyotonia congenita (Wang et al., 2022), sodium channel myotonia (Yadav et al., 2024), and congenital myasthenic syndrome (Liu et al., 2015).

Nav1.5 is mainly expressed in cardiac tissue and can regulate the initial depolarization stage of the cardiac action potential. Nav1.5 deficiency is closely related to a variety of cardiovascular diseases, including long QT syndrome type 3, atrial fibrillation, cardiomyopathy, and Brugada syndrome type 1 (Huang et al., 2019; Salvarani et al., 2023).

Nav1.6 plays an irreplaceable role in the initiation and propagation of neuronal action potentials. It is mainly located at the initial segments and nodes of Ranvier in the axons of neurons in the central and peripheral nervous systems (Bunton-Stasyshyn et al., 2019). Nav1.6 malfunction is closely correlated with many related neurological diseases, including multiple sclerosis (Alrashdi et al., 2019), Alzheimer’s disease (Yuan et al., 2022), lethal movement disorders (Jones et al., 2016), and early infantile epileptic encephalopathy (Lopez-Santiago et al., 2017).

Nociceptive neurons mainly function by Nav1.7, Nav1.8, and Nav1.9. Gene mutations in these channels often lead to loss of pain perception or chronic pain syndrome (Goodwin and McMahon, 2021).

Overall, problems with VGSCs can disrupt the regulatory mechanism of neuronal excitability and trigger a series of physiological abnormalities. In the central nervous system, this dysfunction can affect the accuracy of neural signal transmission, causing a decline in information processing efficiency and accordingly affecting cognitive function and motor coordination ability (Chever et al., 2021; Alsaloum et al., 2025). The situation is different for peripheral neurons. Functional abnormalities in peripheral neurons mainly manifest as sensory disorders and conduction abnormalities (Goodwin and McMahon, 2021; Zhao et al., 2023). As a result, patients may experience symptoms such as hypersensitivity to pain or loss of sensation, and muscle weakness. In terms of cardiac activity, abnormal sodium channels can lead to disorders in the electrical activity of myocardial cells, especially arrhythmia, which can be life-threatening in severe cases (Doundoulakis et al., 2024).

## Voltage-Gated Sodium Channels and Neurodegenerative Diseases

### Voltage-gated sodium channels and familial hemiplegic migraine

FHM is a specific neurological disorder. Although FHM is rare, it shows a clear genetic determination (Pietrobon and Brennan, 2025). It follows an autosomal dominant inheritance pattern; thus, patients have a 50% chance of passing the disease on to their offspring. FHM usually emerges in the early stages of life. The average age of onset is approximately 7 years (Fan et al., 2016). Before the attack, the patient will experience brief neurological dysfunction, the most typical symptom of which is unilateral body movement weakness (hemiplegia). Patients may also exhibit a range of neurological symptoms. Visually, they may experience flickering black dots or a narrowed field of vision. Sensory symptoms can include numbness or tingling in certain parts of the body. Language disorders are also quite common; in mild cases, patients may struggle to find words, while in severe cases, they may be completely unable to speak (Thomsen et al., 2002; Olesen and Steiner, 2004).

**Figure 2:**
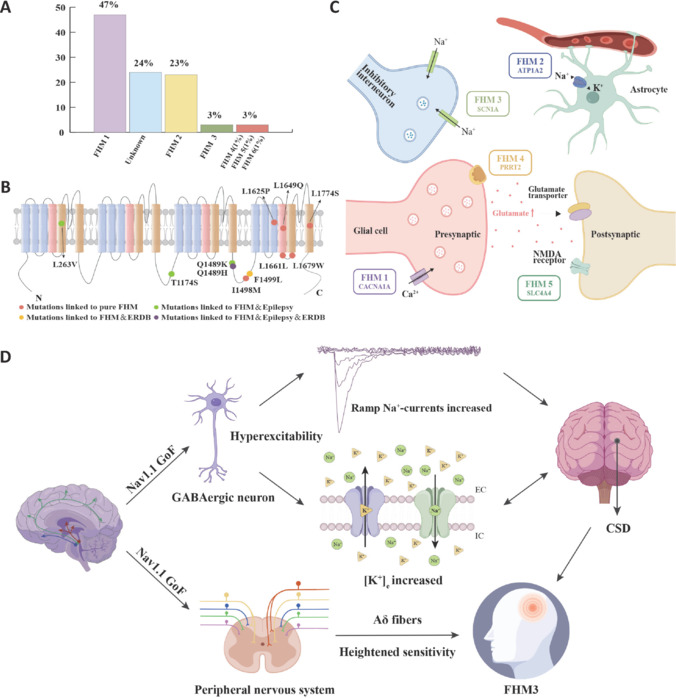
Types of FHM, genetic mechanisms, and pathogenesis of FHM3 induced by Nav1.1 mutations. (A) Relative frequency of identified FHM types (Di Lorenzo et al., 2012). (B) Localization of known FHM3 mutations in the Nav1.1 α-subunit (Dhifallah et al., 2018). (C) Genetic mechanisms of FHM, highlighting glutamatergic synapse proteins encoded by FHM-related genes and their functions (Grangeon et al., 2023). (D) The pathogenesis of FHM3:GoF mutations in Nav1.1 leads to hyperexcitability of GABAergic neurons, causing increased ramp Na^+^ currents and elevated extracellular potassium concentrations ([K^+^]), thereby inducing CSD. Additionally, mutation enhances the sensitivity of Aδ fibers, triggering migraines. A–C are adapted from open access sources, and D is an original image created by the author. ATP1A2: ATPase Na^+^/K^+^ transporting subunit alpha 2; CACNA1A: calcium voltage-gated channel subunit alpha1 A; CSD: cortical spreading depression; ERDB: early repolarization and developmental bradycardia; FHM: familial hemiplegic migraine; GABA: gamma-aminobutyric acid; GoF: gain-of-function; Nav1.1: voltage-gated sodium channel 1.1; NMDA: N-methyl-D-aspartate; PRRT2: proline-rich transmembrane protein 2; SCN1A: sodium channel voltage-gated type 1 alpha subunit.

FHM is primarily divided into three types, namely, FHM1, FHM2, and FHM3. Each type is related to specific gene mutations. The distribution ratio of different FHM subtypes is illustrated in **Figure 2A**, and various mutations and their corresponding symptom spectra are summarized in **Figure 2B**. FHM1 stems from mutations in the *CACNA1A* gene, which encodes the pore-forming α1 subunit of neuronal CaV2.1 calcium channels (Romozzi et al., 2021). FHM1 presents as typical migraine with hemiplegia (including limb paralysis, speech disorders, and visual impairments) lasting from hours to days. FHM2 originates from mutations in the *ATP1A2* gene, which encodes the α2 catalytic subunit of Na^+^/K^+^-ATPase in adult brain astrocytes (Antonaci et al., 2021). It is characterized by migraines accompanied by rapidly progressing neurological deficits, including hemiplegia and disturbances of consciousness, and the symptoms show dynamic progression. FHM3 is associated with missense mutations in the *SCN1A* gene, which encodes the pore-forming subunit of voltage-gated sodium channel Nav1.1 (Dichgans et al., 2005). FHM3 manifests as severe migraines accompanied by transient hemiplegia, aphasia, or other neurological dysfunctions, and is characterized by acute severity but rapid recovery.

While the molecular mechanisms underlying different FHM subtypes vary substantially, they all end up triggering migraine auras by ramping up glutamate signaling and increasing sensitivity to cortical spreading depression (CSD) (Schubert et al., 2018). CSD is a form of brain electrical activity characterized by a slowly propagating, self-sustaining wave of nearly complete depolarization of large populations of neurons in the brain, which silences brain electrical activity for minutes and is closely associated with migraine aura and potentially triggering migraine pain mechanisms (Mathew and Panonnummal, 2022). Specifically, *CACNA1A* gene mutations (FHM1) increase presynaptic calcium influx, promoting excessive glutamate release and significantly enhancing excitatory synaptic transmission (Terpolilli et al., 2022). *ATP1A2* mutations (FHM2) impair synaptic clearance of potassium and glutamate, leading to accumulation of glutamate in the synaptic cleft, activating more N-methyl-D-aspartate (NMDA) receptors, and enhancing neuronal excitability (Dichgans et al., 2005). Meanwhile, *SCN1A* mutations (FHM3) enhance neuronal excitability by altering the function of voltage-gated sodium channel Nav1.1 (Kahlig et al., 2008).

Nav1.1 functional disorders can be categorized as those involving loss of function or excessive enhancement of function. When Nav1.1 function is impaired, the excitability of gamma-aminobutyric acid (GABA)ergic neurons will weaken. This weakens their inhibitory control over the activities of other neurons, disrupts the excitation-inhibition balance in the neural network, and eventually triggers epileptic seizures (Hedrich et al., 2014). For example, c.606del (p.Tyr202Ter) is a truncation mutation that leads to the loss of function of the Nav1.1 protein. This mutation generates a premature termination codon, causing early interruption of protein synthesis. As a result, the generated Nav1.1 protein lacks most of the key functional domains and shows a significant reduction in the number of sodium channels on the cell membrane surface. This change also affects the voltage-sensitive characteristics and activation process of the remaining channels. These alterations prevent inhibitory neurons from effectively generating action potentials. The inhibitory mechanism in neural networks is weakened, and the activity of excitatory neurons becomes uncontrolled. Ultimately, the neural network becomes hyperexcited, triggering epileptic seizures (Jadhav et al., 2024).

In contrast, the function-enhancing mutations of the Nav1.1 channel are mainly related to the pathogenesis of FHM3 (Barros et al., 2014). For example, the c.4495T>C (p.Phe1499Leu) mutation leads to abnormal enhancement of Nav1.1 function (Shao et al., 2018). This mutation alters the inactivation characteristics of the channel. The open state of the channel is prolonged, keeping the neurons in a depolarized state for extended periods. This continuous neuronal activation can cause patients to experience excessive neural excitement and eventually lead to FHM3 (Mantegazza and Broccoli, 2019). This excessive excitement is closely related to the mechanism underlying CSD in the neocortex (Chever et al., 2021). CSD can be caused by pharmacological activation of the Nav1.1 channel or by optically induced overactivity of GABAergic neurons. This process is directly related to the accumulation of extracellular potassium ions. The propagation of CSD waves causes neurons to release a large amount of potassium ions into the extracellular space, further promoting the diffusion of CSD. Notably, neither the synaptic transmission of GABAergic nor glutamatergic neurons is necessary for the initiation of CSD. Thus, the CSD initiation mechanism represents a relatively independent process specific to the neocortex (Chever et al., 2021; Pietrobon and Brennan, 2025).

Some Nav1.1 loss-of-function (LOF) mutations (such as L1649Q) can also trigger FHM3, but these patients do not present with epilepsy or other neurological symptoms (Vanmolkot et al., 2007). This mutation mainly leads to instability or misfolding of the transmembrane segment structure of S4, causing abnormal three-dimensional conformation of Nav1.1 and ultimately resulting in functional loss (Rusconi et al., 2009). The function of the L1649Q mutant Nav1.1 can be partially restored under low-temperature conditions or after the introduction of ankyrin G and calmodulin. Notably, even if the function is only partially restored, the L1649Q mutant may still exhibit enhanced function, leading to excessive excitation of GABAergic neurons (Cestèle et al., 2013). These studies indicate that the main pathogenic mechanism of FHM3 involves Nav1.1 mutations enhancing the excitability of GABAergic neurons, triggering CSD in the neocortex region, and thereby inducing migraine. Although these mechanisms are mainly attributed to the central nervous system, dysfunction of sensory neurons may also be involved in the pathogenesis of migraine. In addition to showing high expression in the central nervous system, Nav1.1 is also distributed in small quantities in myelinated Aδ fibers of the peripheral nervous system. Aδ fibers exhibit high mechanical sensitivity, primarily mediating mechanical nociception and cold sensations (Zhang et al., 2024a). Gain-of-function (GOF) Nav1.1 mutations can significantly enhance Aδ fiber responsiveness to mechanical stimuli (Osteen et al., 2016), indicating that amplification of pain signals through the peripheral nervous system may be another mechanism by which Nav1.1 mutations induce migraines. In summary, the core pathogenic mechanism of FHM3 lies in GOF mutations in Nav1.1 channels promoting GABAergic neuron hyperexcitability, subsequently triggering CSD in neocortical regions, and ultimately leading to the typical clinical manifestations of FHM3 through subsequent activation of meningeal nociceptors (Chever et al., 2021). Simultaneously, these mutations enhance Aδ fiber sensitivity, further exacerbating FHM3 occurrence. These findings highlighting the causes of FHM3 are paving the way for new targeted treatments.

Two strategies are used to treat FHM: prevention and management of acute attacks. Preventive treatment is usually adopted when the attack frequency exceeds once every 2 months or seriously affects the patient’s quality of life (Pelzer et al., 2013). Preventive intervention often uses drugs such as flunarizine, lamotrigine, and valproic acid (VPA). These drugs essentially alleviate symptoms by regulating neuronal excitability and the CSD threshold. Flunarizine simultaneously inhibits neuronal sodium channels and calcium channels, reduces cortical hyperexcitability, and also increases the CSD threshold (Ye et al., 2011; De Cunto et al., 2012). The therapeutic effect of lamotrigine is primarily mediated by blocking sodium channels and N-/P-/Q-type calcium channels, thereby inhibiting the release of glutamate (Athwal and Lennox, 1996; Chen et al., 2001). Sodium valproate enhances neuronal inhibitory effects by inhibiting GABA transaminase, activating glutamate decarboxylase, and blocking sodium channels (Cutrer et al., 1997; Evers et al., 2009). During acute attacks, intravenous injection of verapamil improves symptoms by blocking L-type calcium channels; this drug can also block P-/Q-type channels at high doses (Yu and Horowitz, 2003). Acetazolamide stabilizes abnormal ion channels by inhibiting carbonic anhydrase and reducing serum bicarbonate levels (Strupp et al., 2007). In patients with persistent headache, triptan drugs can be considered, although their use is controversial. While *SCN1A* gene mutations have been confirmed as the cause of FHM3, specific treatment options for these patients are still insufficient. Most clinical treatments still follow the traditional FHM treatment strategy (Pelzer et al., 2013).

Recent studies have made substantial progress in therapeutic research on FHM3. Clinical observations have shown that carbamazepine can significantly alleviate the symptoms of some patients with FHM3 carrying *SCN1A* GOF mutations. This antiepileptic drug can effectively inhibit excessive sodium ion influx caused by sodium channel hyperfunction, thereby reducing the frequency and severity of attacks in patients with FHM3. Notably, the effect of carbamazepine is not completely selective; it simultaneously affects multiple sodium channel subtypes (including *SCN2A* and *SCN3A*), which may explain the individual differences observed in the treatment response (Brunklaus et al., 2020). The novel late sodium current inhibitor GS967 has become a promising therapeutic candidate for FHM3 (Anderson et al., 2017). This compound exhibits selective binding and stability to the inactivated state of the Nav1.1 channel, effectively inhibiting the persistent sodium current induced by FHM3 mutations, while significantly slowing down the recovery of the channel from inactivation. In comparison with conventional SCBs, GS967 shows a more specific effect. It can selectively block the pathological persistent current caused by FHM3 mutations while having a relatively small impact on normal sodium channel function. The therapeutic effects of GS967 targeting FHM3 have been widely evaluated before clinical practice (Barbieri et al., 2019). In the future, methods for restoring the function of Nav1.1 or regulating its abnormal activities are expected to yield better therapeutic effects and clinical improvements in patients with FHM3, and simultaneously provide new ideas for research on related neurological diseases.

### Voltage-gated sodium channels and epilepsy

**Figure 3:**
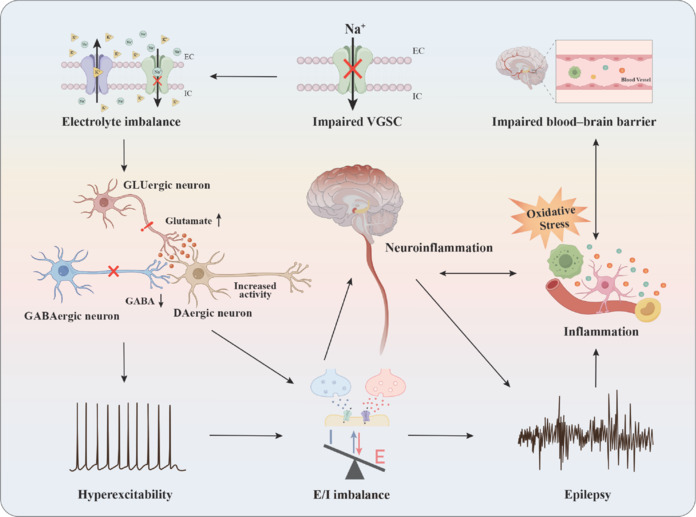
The mechanism linking sodium channel dysfunction to epilepsy. Damage to VGSCs results in electrolyte imbalances that disrupt neuronal homeostasis. This disruption leads to increased glutamate release and decreased GABA activity, which in turn causes neuronal hyperexcitability and disturbs the balance between excitatory (E) and inhibitory (I) signals, ultimately resulting in neuroinflammation. Furthermore, oxidative stress exacerbates this situation by damaging the blood‒brain barrier and promoting inflammation, which can ultimately trigger epileptic seizures. DAergic: Dopaminergic; EC: extracellular; GABA: gamma-aminobutyric acid; GLUergic: glutamatergic; IC: intracellar; VGSC: voltage-gated sodium channel.

Epilepsy is a common chronic neurological disorder primarily characterized by abnormal, excessive, or synchronous neuronal activity in the brain (Magro, 2025). This neuronal activity exhibits brief but very prominent signs and symptoms, featuring chronic and recurrent characteristics (Jiao et al., 2025; Liu et al., 2025). The annual incidence rate of epilepsy is approximately 61.4 cases per 100,000 people, with 51 million cases of active epilepsy worldwide. The prevalence of epilepsy in low- and middle-income countries is much higher than that in high-income countries (Asadi-Pooya et al., 2023). Although some progress has been made in treatment using antiepileptic drugs, more than one-third of the patients still develop drug resistance, and the existing drugs may induce relatively serious adverse reactions. This finding highlights the substantial unresolved challenges in the treatment of epilepsy (Klein et al., 2024). The high incidence of epilepsy and the difficulty in its treatment have led to an urgent need to clarify its pathogenesis and formulate more effective treatment strategies. More than 100 different genes have shown genetic alterations related to the causes of epilepsy. Notably, among these identified genes, one-third encode neuronal ion channels, particularly highlighting the dysfunction of VGSCs. Disorders of VGSCs have been proven to play a very fundamental and crucial role in the pathophysiology and manifestation of epileptic seizures (Yu et al., 2024). Dysfunction of VGSCs can disrupt neuronal homeostasis through a dual mechanism: on one hand, it increases the release of the excitatory neurotransmitter glutamate and reduces the activity of γ-aminobutyric acid, thereby causing neuronal hyperexcitability; on the other hand, it disrupts the integrity of the blood–brain barrier, and thereby triggers and intensifies the neuroinflammatory response (Phoswa and Mokgalaboni, 2023). The imbalance between excitatory (E) and inhibitory (I) neurotransmission, coupled with persistent inflammatory responses, forms a self-sustaining pathological cycle that ultimately leads to the occurrence and development of epilepsy (**Figure 3**). Four sodium channel genes, i.e., *SCN1A*, *SCN2A*, *SCN3A*, and *SCN8A*, are mainly expressed in the brain (Johannesen et al., 2022; Chung et al., 2023; Martín et al., 2025), and a sodium channel gene mainly expressed in the heart (*SCN5A*) is associated with various types of epilepsy (Remme, 2023). A comprehensive understanding of the influence of these gene mutations on neuronal function will provide new strategies and insights for the early diagnosis and treatment of epilepsy.

#### Sodium channel voltage-gated type 1 alpha subunit-related epilepsy syndromes

*SCN1A* has been confirmed to be the most common gene causing epilepsy (Symonds et al., 2019). Studies have shown that mutations in this gene can lead to various clinical phenotypes, including febrile convulsions (Damiano et al., 2020), genetic epilepsy with febrile seizures plus (Cornejo-Sanchez et al., 2022), and DS (Steel et al., 2017) and early infantile epileptic encephalopathy (Nájera-Chávez et al., 2023). Among them, DS is the most common disease related to *SCN1A* mutations (Valassina et al., 2022). DS is a relatively rare developmental epileptic encephalopathy, also known as infantile severe myoclonic epilepsy. This disease usually manifests within the first year after birth. Its clinical manifestations are initially febrile convulsions, which rapidly progress to refractory spontaneous epilepsy. Patients with this disease usually experience a variety of systemic symptoms, such as cognitive dysfunction, social problems, hyperactivity, circadian rhythm, and sleep disorders (Dravet, 2011). The genetic mechanism underlying DS has been widely studied, and more than 80% of patients have haploinsufficiency due to mutations in the *SCN1A* gene (Myers, 2023). However, a few mutations in Nav1.1 functions can also lead to DS. For example, p.Ala1772Val and p.Phe1797Ser have been predicted to be GOF mutations. These mutations abnormally enhance the activity of the Nav1.1 channel, which may involve a reduced activation threshold, prolonged opening time, or impaired inactivation process (Negi et al., 2024). The Nav1.1 channel encoded by *SCN1A* is mainly expressed in inhibitory neurons of the brain and is also expressed in GABAergic interneurons and parvalbumin (PV) interneurons (Yamagata et al., 2023). These Nav1.1 channels are crucial for suppressing the generation of neuronal action potentials and also play a key role in maintaining normal neuronal discharge patterns. Damage to the Nav1.1 channel reduces the activation ability of inhibitory neurons. The resultant inhibitory neural transmission in the brain can cause an imbalance in the excitation-inhibition network, after which the clinical symptoms of DS eventually emerge (Stein et al., 2019). Studies have shown that in the early stage of the disease (P11–21) in patients with DS, dysfunction of Nav1.1 can lead to temporary damage to PV interneurons, triggering epileptic seizures (Favero et al., 2018; Mich et al., 2025). This is consistent with the initial stage of SCN1A expression. However, after P30, the excitability of PV interneurons will return to normal. Temporary injury of PV interneurons caused by abnormal Nav1.1 function may trigger epileptic seizures, but it is not the main cause of chronic epilepsy in DS (Favero et al., 2018). Early PV interneuron dysfunction may only play an “initiating” role. What truly affects the formation of late-stage stable chronic epilepsy is very likely the compensatory reorganization of the cortical neural circuit in the early developmental stage of DS (Valassina et al., 2022). Overall, dysfunction of the Nav1.1 channel can prevent inhibitory neurons from generating normal action potentials, weaken inhibitory neural transmission, and disrupt the balance of the excitation-inhibition network. This imbalance can not only directly trigger epileptic seizures, but can also lead to pathological network remodeling, posing significant challenges to the treatment of DS.

The standard treatment for DS mainly relies on antiepileptic drugs to control symptoms. First-line therapy is usually used in combination with VPA and clobazam (CLB) (Musto et al., 2020). VPA stabilizes neuronal membranes by enhancing GABA-mediated inhibitory effects and blocking voltage-dependent sodium channels, while CLB, a benzodiazepine drug, can enhance the inhibitory neurotransmission of GABA type A (GABAA) receptors. SCBs such as phenytoin, lamotrigine, carbamazepine, and oxcarbazepine are usually regarded as contraindications. However, some patients with DB have shown positive responses to these drugs. This finding indicates that these drugs may function not merely by blocking sodium channels (Dalic et al., 2015; Snoeijen-Schouwenaars et al., 2015; Zographos et al., 2022; Myers, 2023).

The Food and Drug Administration (FDA) has approved several new drugs with different mechanisms of action for DS. Stiripentol (STP), as a second-line therapeutic drug, mainly exerts its effect by regulating the highly expressed GABAA receptor (α3 subunit) in the developing brain. Thus, initiation of STP treatment before adolescence may have a better effect. In addition, STP can increase the blood concentration of norclobazam, the active metabolite of CLB, and shows a synergistic effect when used in combination with VPA and CLB (Alsaloum et al., 2025). Cannabidiol (CBD) has multiple mechanisms of action and can effectively reduce convulsive and, potentially, non-convulsive seizures. It particularly exerts its effects through interactions with CLB and its active metabolites. Fenfluramine, an amphetamine derivative, mainly regulates the serotonin pathway and is used as an adjuvant in clinical trials (Musto et al., 2020).

The existing approved drugs provide symptom relief mainly by reducing the frequency of epileptic seizures. However, they have limited effects on improving other symptoms such as cognitive impairment, abnormal gait, and sleep disorders. Therefore, development of treatment strategies directly targeting Nav1.1 dysfunction has become increasingly important. Recent studies on genetic therapy for DS have focused on restoring Nav1.1 channel expression levels, enhancing normal *SCN1A* allele expression at transcriptional and post-transcriptional levels to compensate for functional allele deficiency. Nicholas Valassina successfully restored *Scn1a* gene expression in conditional rescue mouse models through systemic delivery of adeno-associated virus (AAV) expressing Cre recombinase. Notably, restoring Nav1.1 expression levels post-symptom onset (P30) significantly improved both spontaneous and heat-induced seizures while also ameliorating behavioral abnormalities, including hyperactivity, social deficits, and cognitive dysfunction. Remarkably, even in adult DS mice (P90) with prolonged seizure history, Nav1.1 expression restoration completely suppressed seizures. Transcriptomic analysis further revealed that this therapeutic approach reversed alterations of gene expression in the cortex and hippocampus, particularly those associated with astrocytic activation (Valassina et al., 2022). Yuan et al. (2024) recently studied an innovative therapeutic strategy using STK-001 (ASO-22), an antisense oligonucleotide targeting exon 20N of the human *SCN1A* gene. As a therapeutic candidate in clinical development, STK-001 uses TANGO technology to suppress the inclusion of exon 20N containing the premature termination codon, effectively increasing the expression levels of Scn1a transcripts and Nav1.1 protein. Studies have shown that a single intraventricular injection of STK-001 to DS mice can significantly reduce the occurrence of epileptic seizures and accidental deaths in the mice (Miziak and Czuczwar, 2021; Yuan et al., 2024). In mechanism studies using ASO-84 (an alternative to ASO-22) (Hill and Meisler, 2021; Wengert et al., 2022), a single injection of ASO-84 administered on the second day after birth restored the action potential discharge pattern and sodium current density of cortical PV interneurons in DS mice. GABAergic signal transduction was restored, normalizing the inhibitory postsynaptic current frequency in cortical pyramidal neurons. These findings provide crucial theoretical support for the treatment of *SCN1A*-related DS using antisense oligonucleotides such as ASO-22 (Yuan et al., 2024). Treatment strategies for DS have evolved from traditional symptom management to gene-based precision medicine. Among the innovative therapeutic agents under development, STK-001 (ASO-22) has entered clinical trials, showing promising therapeutic potential. *ETX101*, developed by Encoded, is another gene therapeutic agent. It uses the AAV9 vector to deliver engineered transcription factors and improves the symptoms of DS by enhancing the expression of the *SCN1A* gene (Myers, 2023). Both of these new genetic therapies have received FDA orphan drug for rare pediatric disease designations, bringing new hope to patients with DS.

#### Sodium channel voltage-gated type 2 alpha subunit-related epilepsy syndromes

*SCN2A* is the second-most common epileptogenic gene after *SCN1A*, which was the first reported epileptogenic gene (Heyne et al., 2019). The *SCN2A* gene encodes the Nav1.2 channel protein. Nav1.2 mainly exists in the axons of excitatory neurons and the initial segments of unmyelinated axons, and is distributed in regions such as the cerebral cortex, hippocampus, and striatum (Zeng et al., 2022). It is crucial for the generation and propagation of action potentials. During the early development stage of neurons, it is the only voltage-dependent sodium channel gene expressed. Researchers have identified many variations of *SCN2A*. These include missense variations, frameshift deletions or insertions, nonsense variations, and splicing site variations. Variations can increase or decrease neuronal excitability by altering the function of Nav1.2, leading to various clinical manifestations. Missense variations are the most common type among *SCN2A* variations (Yamagata et al., 2017). For example, the p.Ala1316Val mutation is a missense mutation in the *SCN2A* gene. This mutation makes the sodium channel easier to open and more difficult to close and also lowers the voltage threshold required to activate it. As a result, neurons are abnormally excited, which leads to uncontrollable epilepsy. Children with this mutation not only experience frequent seizures, but also often show severe developmental delays and abnormalities in the white matter (Epifanio et al., 2021). Epilepsy related to *SCN2A* variations usually occurs in early childhood. Its phenotypic range is very wide, ranging from benign self-limiting epilepsy to developmental epileptic encephalopathy. GOF mutations of the *SCN2A* gene are key pathogenic factors for a variety of neurological diseases. These diseases include benign familial neonate-infant epilepsy, Ohtahara syndrome, early-onset infantile epileptic encephalopathy, and early-onset developmental epileptic encephalopathy (Wolff et al., 2017). These mutations exert their effects by enhancing the activity of the Nav1.2 sodium channel, causing increased sodium ion influx, enhanced neuronal depolarization, and prolonged channel inactivation time, and ultimately resulting in abnormal neuronal excitability. Patients with GOF mutations of *SCN2A* usually experience their first episode within 3 months after birth. They exhibit severe global developmental delay, intellectual disability, and motor disorders, accompanied by persistent refractory epileptic seizures. These patients have a poor prognosis, which manifests as an increased risk of progressive dysfunction and early death (Scheffer et al., 2017). In contrast, the LOF variation of *SCN2A* usually presents as a late-onset (> 3 months) phenotype. These variations are associated with infantile spasms, West syndrome, late-onset myoclonic-atonic epilepsy, developmental encephalopathy, ASD, or intellectual disability (Zeng et al., 2022). These mutations reduce the function of the Nav1.2 channel and weaken the excitability of neurons, generating diverse clinical phenotypes.

The treatment of epilepsy caused by hereditary *SCN2A* variations mainly relies on SCBs. These drugs include carbamazepine, phenytoin, oxcarbazepine, lamotrigine, lidocaine, and mexiletine (Musto et al., 2020). Among them, oxcarbazepine is the most common prescription drug. Studies have shown that although oxcarbazepine is the most effective antiepileptic treatment option, its epilepsy control rate is still less than 30%. SCBs are mainly effective for patients with seizure onset before 3 months of age, while they may aggravate epileptic seizures in patients with onset after 3 months (Wolff et al., 2017; Yuan et al., 2023). Zeng et al. (2022) also reported similar findings. They suggested that SCBs should not be used in patients who developed the disease after the age of 1 year. For patients who develop the disease before 1 year of age, treatment with SCBs can be attempted with caution and due consideration of the possible risk of aggravating epileptic seizures. In addition to SCBs, VPA has also demonstrated a moderate antiepileptic effect, with an epilepsy control rate of approximately 22%. Although the epilepsy control rate of VPA is slightly worse than that of oxcarbazepine, VPA shows a lower risk of aggravating epilepsy. In addition, topiramate, levetiracetam, and phenobarbital can also control epileptic seizures in some patients, although their overall effect is limited. These oral medications each have their own characteristics: SCBs, which are represented by oxcarbazepine, have the best effect on early-onset patients, but their overall control rate is low, and their use in late-onset cases can aggravate symptoms. Although VPA has a relatively low effect, it causes fewer adverse reactions and is a relatively safe alternative. Other drugs such as topiramate have limited effects, but they have expanded the clinical treatment options for these patients. In addition to conventional antiepileptic drugs, methods to suppress epilepsy at the gene expression level are also under investigation. Antisense oligonucleotide therapy can specifically inhibit abnormal *SCN2A* gene expression, significantly reduce the seizure frequency in epilepsy models caused by *SCN2A* GOF mutations, and prolong the lifespan of model mice (Goldberg, 2021). At present, the antisense oligonucleotide drug PRAX-222 developed by Praxis Precision Medicines has entered phase II clinical trials. This drug can selectively reduce the expression level of Nav1.2 in patients with *SCN2A* GOF epilepsy, demonstrating significant therapeutic effects in animal models and has been recognized as an orphan drug for a rare pediatric disease by the FDA. By specifically inhibiting the expression of abnormal *SCN2A* genes, antisense oligonucleotide therapy provides an innovative genetic intervention method for the treatment of *SCN2A*-related epilepsy.

#### Sodium channel voltage-gated type 3 alpha subunit-related epilepsy syndromes

*SCN3A* encodes the Nav1.3 channel protein, and this subunit is highly expressed in the embryonic brain (Cheah et al., 2013). Neurodevelopmental disorders related to *SCN3A* present a unique phenotypic spectrum. Some patients present with developmental and epileptic encephalopathy (DEE), which may or may not be accompanied by cortical malformations. Others show mild focal epilepsy along with cortical malformations. Some patients only show isolated cortical malformations and no epileptic manifestations (Zaman et al., 2020). Both GOF and LOF mutations in the *SCN3A* gene can lead to the occurrence of epilepsy. Early-onset epileptic encephalopathy is usually associated with a significant functional enhancement of *SCN3A*. This mainly manifests as a significant increase in persistent sodium current, enhancing the excitability of neurons and thereby promoting epileptic seizures. In contrast, milder symptoms may be associated with Nav1.3 LOF or mild functional enhancement. Notably, the commonly used antiepileptic drug valproate can alleviate epileptic seizures by effectively reducing the expression level of *Scn3a* in mouse models (Tan et al., 2016). In addition, SCBs such as lacosamide, carbamazepine, topiramate, and phenytoin can alleviate epileptic symptoms by reducing the GOF effects of *SCN3A*. However, in comparison with other sodium channel genes (such as *SCN1A*, *SCN2A*, and *SCN8A*), gene therapy research targeting *SCN3A* is relatively scarce, limiting the development of precise treatment strategies for epilepsy related to *SCN3A*.

#### Sodium channel voltage-gated type 8 alpha subunit-related epilepsy syndromes

The Nav1.6 channel encoded by the *SCN8A* gene is the most abundant sodium channel subtype in the central nervous system. It is mainly distributed in the axon initial segment and nodes of Ranvier. At the axon initial segment, the Nav1.6 channel is involved in the generation of action potentials. In the nodes of Ranvier, these channels mediate saltatory conduction, ensuring that nerve impulses can be transmitted rapidly (Lopez-Santiago et al., 2017). Mutations in the *SCN8A* gene can cause various types of epileptic symptoms with varying degrees of severity. These manifestations can be classified into multiple categories: The first is destructive developmental epileptic encephalopathy, wherein patients present with severe intellectual disability, regression of motor function, gradual deterioration of vision, and refractory epilepsy. Such patients have a poor prognosis and a high early mortality rate. The second category is treatable epilepsy, wherein patients have mild intellectual disability and some neurological abnormalities, such as ataxia, hypotonia, tremors, and myoclonus. The third category is benign familial infantile epilepsy, in which patients may develop movement disorders in the following years, especially paroxysmal dyskinesia. The last category of patients do not have epilepsy but have cognitive and/or behavioral disorders or motor disorders (Musto et al., 2020).

Similar to *SCN2A* mutations, *SCN8A* GOF mutations can lead to aggravation of epilepsy (Veeramah et al., 2012; Estacion et al., 2014). LOF mutations of *SCN8A* usually cause intellectual disability or autism, but do not lead to epileptic seizures (Trudeau et al., 2006; Wagnon et al., 2017). However, in a few cases, such mutations may also cause epilepsy and intellectual disability simultaneously (Blanchard et al., 2015). Although epilepsy caused by mutations in *SCN8A* and *SCN2A* shows similarities, obvious differences have also been reported. *SCN2A*-related epilepsy usually manifests after birth or in early infancy (typically within 3 months), and more common accompanying symptoms are delayed language development and sensory integration dysfunction. Furthermore, epilepsy related to *SCN8A* mutations usually occurs at 4–6 months and is often accompanied by motor dysfunction and progressive visual impairment. The existing research has provided an in-depth understanding of the mechanisms by which *SCN8A* mutations cause epilepsy. Tidball et al. (2020) studied three patients carrying different SCN8A variations and found that the proportion of persistent sodium currents in cortex-like neurons of two of the patients increased, while the neurons of the third patient showed an increase in resurgent sodium currents. The neurons of all patients with epilepsy showed slowed action potential repolarization and reduced length of axon initial segments, accompanied by increased network burst activity (Tidball et al., 2020). The study by Liu et al. (2019) indicates that *SCN8A* mutations that increase neuronal discharge activity can lead to epilepsy phenotypes ranging from mild to severe, possibly with or without intellectual disability. Conversely, the LOF mutations of *SCN8A* that reduce neuronal discharge activity mainly manifest as developmental delay, intellectual disability, or autism, but do not result in epileptic seizures. In conclusion, the GOF mutation of *SCN8A* typically increases the persistent sodium current, prolongs the repolarization time of the action potential, and enhances the resurgent sodium current. These changes maintain neurons in a depolarized state for a long time and increase their excitability. Such mutations also alter the spontaneous discharge patterns of neurons, which manifests as more frequent and intense network burst activities. However, certain mutations can significantly slow down the inactivation of sodium ion channels. Although they manifest as GOF mutations at the molecular level, they may lead to a depolarization blockade at the neuronal level, reduce neuronal discharge, and show a reduction in the length of axon initial segments, which may be a compensatory response to hyperexcitability.

Given the complex clinical phenotypes and pathogenic mechanisms of *SCN8A*-related epilepsy, the treatment strategies demonstrate diverse characteristics. In mild phenotypes, seizures may resolve spontaneously or respond well to single SCBs and other AEDs. However, in severe phenotypes, particularly DEE, although conventional SCBs (such as phenytoin, carbamazepine, oxcarbazepine) show some efficacy, supra-therapeutic doses are typically required for seizure control. For drug-resistant patients, various adjunctive therapeutic options based on different mechanisms of action are available. These include zonisamide, which modulates both voltage-gated sodium channels and T-type calcium channels through dual mechanisms; STP, which enhances inhibitory neurotransmission by positively modulating the GABAA receptor α3 subunit; lacosamide, which reduces abnormal discharges by selectively enhancing the slow inactivation of sodium channels; rufinamide, which acts through modulation of sodium channel kinetics and enhancement of GABAergic transmission; and perampanel, which regulates glutamatergic transmission as a selective non-competitive α-amino-3-hydroxy-5-methyl-4-isoxazolepropionic acid (AMPA) receptor antagonist (Alsaloum et al., 2025).

Clinical observations indicate substantial therapeutic benefits of these medications in some patients with drug-resistant DEE. Additionally, benzodiazepines, as positive allosteric modulators of GABAA receptors, hold value in acute management of seizure clusters, prolonged seizures, and status epilepticus (Musto et al., 2020). Recent studies have identified potential therapeutic agents with relative selectivity for Nav1.6 channels. The novel selective Nav1.6 inhibitors Prax330 and NBI-921352 show particular promise. Prax330 specifically inhibits persistent and resurgent sodium currents in Nav1.6 channels, demonstrating effective antiepileptic effects in mouse models of *SCN8A* epilepsy (Wengert et al., 2019). Johnson et al. (2022) evaluated the efficacy of NBI-921352, the first highly selective Nav1.6 inhibitor that has entered phase II clinical trials, in *SCN8A* developmental and epileptic encephalopathy (*SCN8A*-DEE) and adult focal seizures. Through high-throughput drug screening, Atkin et al. (2018) identified three candidate drugs from 1320 marketed medications showing significant inhibitory effects on mutant Nav1.6 channels: carvedilol (β-blocker originally used for congestive heart failure); amitriptyline (tricyclic antidepressant, 5-hydroxytryptamine-norepinephrine reuptake inhibitor); and nifedipine (calcium channel blocker originally used for hypertension). *In vitro* experiments confirmed that these three drugs effectively inhibit abnormal neuronal excitability caused by the Nav1.6 R1872Q mutation, providing new options for precision treatment of *SCN8A*-related epilepsy (Atkin et al., 2018). Patel et al. (2016) further discovered that cannabidiol preferentially targets peak transient currents produced by wild-type Nav1.6 channels as well as abnormal resurgent and persistent currents generated by mutant Nav1.6 channels. Thus, resurgent currents may serve as a promising therapeutic target for epilepsy syndromes. However, the existing pharmacological treatments show limited ability to fundamentally correct Nav1.6 dysfunction. Given these limitations, researchers have begun exploring direct intervention strategies targeting the *SCN8A* gene. Gene therapy approaches, such as lentiviral-mediated Cre injection to knock down *Scn8a* gene expression, have shown positive effects on reducing seizure frequency in animal models (Makinson et al., 2014). Intracerebroventricular administration of *Scn8a* antisense oligonucleotides reducing Nav1.6 expression by 50% can also decrease seizures and extend survival time (Lenk et al., 2020), although antisense oligonucleotide therapy shows limitations associated with the repeated administrations and difficulties in controlling the effects of gene expression. Recent research achieving allele-specific editing to selectively inactivate pathogenic alleles has shown effective improvement in epileptic symptoms. By specifically disrupting the reading frame of pathogenic transcripts, this strategy produces frameshift mutations in 25%–33% of transcripts throughout the brain. Even this relatively modest improvement in editing efficiency significantly reduces mortality rates and seizure frequency in animal models (Yu et al., 2024). In conclusion, gene therapy, through its advantages of high specificity and durability along with the rapid regulatory effects of novel selective Nav1.6 inhibitors, provides more comprehensive and personalized therapeutic strategies for *SCN8A*-related epilepsy.

#### SCN5A-related epilepsy syndromes

In addition to the four well-characterized voltage-gated sodium channels that play pivotal roles in epileptogenesis within the central nervous system, the Nav1.5 voltage-gated sodium channel encoded by the *SCN5A* gene has emerged as a subject of substantial scientific inquiry. This channel, which is predominantly expressed in cardiomyocytes, exerts a deterministic influence on cardiac action potential generation and rhythm initiation (Jiang et al., 2020). Extant literature has mechanistically implicated both GOF and LOF variants of *SCN5A* in the pathogenesis of Brugada syndrome and long QT syndrome type 3 (Hummel et al., 2013; Shen et al., 2024). A recent seminal investigation elucidated the novel functional implications of *SCN5A* in epilepsy and its severe sequelae: over 10% of the cases of sudden unexpected death in epilepsy (SUDEP) harbor mutations in both SCN5A and another critical gene, *KCNH2*. SUDEP, one of the most devastating complications of epilepsy, exhibits a mortality rate of up to 18%, predominantly affects young individuals, and has emerged as the principal cause of epilepsy-related mortality (Zhang et al., 2022). Ongoing in-depth research has revealed that the pathophysiological mechanism of SUDEP includes the synergistic effects of abnormalities in epilepsy-related genes and variations in arrhythmia-related genes (such as *SCN5A*). Arrhythmia and respiratory arrest constitute the main pathways of death. Notably, the expression of the Nav1.5 channel is not limited to cardiac tissue. It is also prominently present in the limbic forebrain of mammals, where it plays a crucial regulatory role in neuronal discharge patterns (Li et al., 2023). Furthermore, the functional expression of Nav1.5 channels in astrocytes indicates that these channels play an important role in maintaining the balance of neuronal electrical activity. Dysfunction of these channels may increase the risk of epileptic seizures (Johnson et al., 2009). Therefore, Nav1.5 dysfunction may increase the risk of SUDEP through a dual mechanism. This mechanism simultaneously affects cardiac rhythm and neuronal function and may cause severe arrhythmias during or after epileptic seizures (Soh et al., 2022). The treatment methods for SUDEP caused by Nav1.5 dysfunction are still in the early stage of development. Relevant research is still lacking. However, considering the fundamental role of *SCN5A* in regulating the electrophysiological activities of the heart and neurons, it has considerable therapeutic potential as a molecular target for SUDEP intervention. This is worthy of comprehensive scientific research and clinical exploration.

Epilepsy caused by voltage-gated sodium channel dysfunction has obvious subtype specificity. The Nav1.1 channel encoded by the *SCN1A* gene is mainly expressed in inhibitory interneurons. The loss of such channel functions weaken inhibitory neuronal transmission and disrupt the excitation-inhibition balance in neural networks. In contrast, the Nav1.2 and Nav1.6 channels encoded by *SCN2A* and *SCN8A* are mainly expressed in excitatory neurons. Function-enhancing mutations of these channels can increase the excitability of neurons and trigger severe early-onset epilepsy. Their LOF mutations are mainly associated with cognitive impairment and ASD. The expression level of the Nav1.3 channel encoded by *SCN3A* is very high during the embryonic development period. Functional abnormalities of this channel may trigger early-onset epileptic encephalopathy by increasing persistent sodium current, or manifest as a milder epileptic syndrome. Furthermore, mutations in the *SCN5A* gene, which are mainly expressed in cardiomyocytes, not only affect the electrophysiological functions of the heart but may also increase the risk of sudden death in epilepsy patients by acting on neurons and astrocytes in the limbic forebrain region. These findings have greatly deepened our understanding of the crucial role of voltage-gated sodium channels in regulating neuronal excitability and maintaining the homeostasis of neural networks. Thus, these results provide a fundamental theoretical foundation for the development of targeted therapeutic strategies.

### Voltage-gated sodium channels and autism spectrum disorder

**Figure 4:**
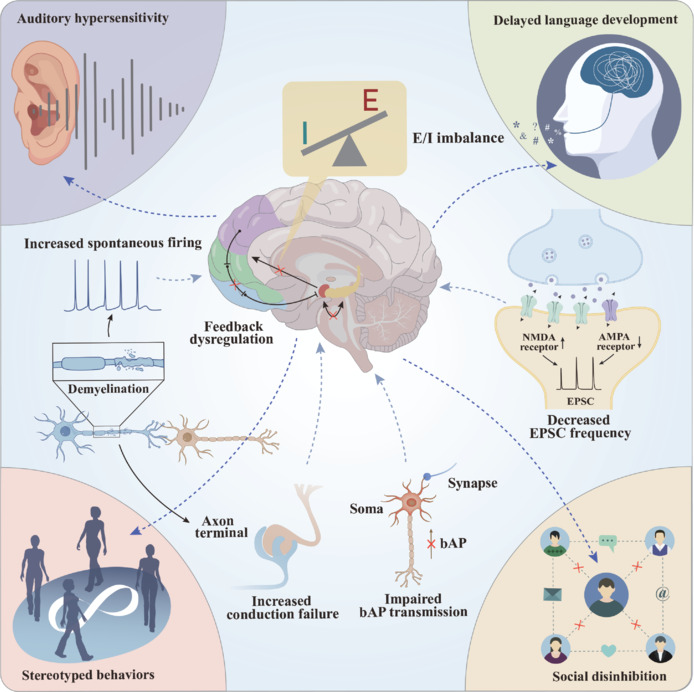
Mechanism of sodium channel dysfunction in ASD. Dysfunction of voltage-gated sodium channels causes myelin damage, bAP conduction failure, and synaptic impairment, disrupting internal feedback and the E/I balance in the brain. These changes lead to sensory sensitivity, delayed language development, repetitive behaviors, and social deficits—hallmark features of ASD. AMPA: α-Amino-3-hydroxy-5-methyl-4-isoxazolepropionic acid; ASD: autism spectrum disorder; bAP: back-propagating action potential; E/I: excitatory/inhibitory; EPSC: excitatory postsynaptic current; NMDA: N-methyl-D-aspartate.

ASD is a complex neurodevelopmental disorder that severely affects social communication and behavioral patterns. The prevalence of ASD has increased substantially in recent decades (Martinez and Peplow, 2025). Epidemiological investigations show that the diagnosis rate of ASD has increased significantly: The prevalence in the United States rose from 1.1% in 2008 to 2.3% in 2018, and the reported rates in other developed countries were also between 1.5% and 2.3% (Lord et al., 2018; Hirota and King, 2023). The clinical manifestations of ASD include sensory hyperresponsiveness, delayed development of the language system, and obvious stereotyped behaviors and social disorders. Patients often have a relatively high risk of comorbidities as well. Notable comorbidities include depression (20%), anxiety disorders (11%), and sleep disorders (13%), whose incidences in patients with ASD are significantly higher than those in the general population (Hirota and King, 2023; Angel et al., 2025; **Figure 4**). ASD shows a clear association with epileptic manifestations. More than 30% of individuals with ASD experience epileptic seizures during infancy or childhood. However, in many cases, epileptic activity weakens or completely disappears in the later developmental stages (Kaczmarek, 2019). High-throughput sequencing has identified 65 risk genes related to ASD (Sanders, 2015). These genes can be divided into two major categories: chromatin modification factors and genes that support synaptic function (Spratt et al., 2019). Among the genes related to synaptic function, protein truncation and missense variations of *SCN2A* have been identified as the main variations in children with ASD. The *SCN2A* gene is widely regarded as one of the major genes closely related to the pathogenesis of ASD (Ma et al., 2022).

The *SCN2A* gene encodes the voltage-gated sodium ion channel Nav1.2. This type of sodium ion channel is widely expressed in the central nervous system. It plays a key role in axonal excitability during the early developmental stage, and is also crucial for action potential backpropagation, dendritic excitability, synaptic transmission, and synaptic plasticity of mature pyramidal neurons (Wang et al., 2021). Different functional variants of *SCN2A* are associated with distinct neurodevelopmental disorders. The GOF mutation of *SCN2A* is significantly correlated with infantile epilepsy of different severities (Spratt et al., 2019). The heterozygous LOF variant of *SCN2A* is significantly associated with autism and intellectual disability by reducing or eliminating channel function (haploinsufficiency), while homozygous deletion leads to embryonic death. Since complete deletion of *SCN2A* would lead to fatal consequences, researchers ingeniously utilized the *Scn2a*^+/–^ mouse model (knocking out one *Scn2a* gene allele) to explore the potential pathological mechanisms underlying autism. These *Scn2a*^+/–^ heterozygous deficient mice exhibited several common characteristics of schizophrenia and autism models, particularly mild social behavior deficits, enhanced fear conditioned reflexes, and impaired fear extinction (Tatsukawa et al., 2019). In addition to behavioral abnormalities, *Scn2a* haploinsufficiency also causes a variety of electrophysiological changes, especially in dendritic excitability. In mature brains, Nav1.2 mainly supports backpropagating action potentials (bAPs) within the dendrites of pyramidal cells in the neocortex (Larkum et al., 1999b). These bAPs promote dendritic depolarization and can produce dendritic spikes when paired with local synaptic stimuli or during high-frequency bursts (Larkum et al., 1999a). Spratt et al. (2019) found that during the mature stage, the bAP ability of *Scn2a*^+/–^ mice was significantly reduced by the loss of Nav1.2 channel function in the cell body and dendrites. Although the initiation and propagation of the action potential remained intact, the dendrite depolarization ability was greatly reduced, resulting in a significant decrease in calcium ion influx into the distal dendrites. Regarding axon function, no significant changes have been reported in presynaptic parameters such as calcium ion transients at the axon terminals and the ratio of paired pulses. However, in terms of synaptic function, the decrease in the frequency of miniature excitatory postsynaptic current (mEPSC) and the reduction in the ratio of NMDA to AMPA indicate an increase in the proportion of “silent synapses” (Spratt et al., 2019).

Overall, mutations or defects in *SCN2A* lead to dysfunction of the Nav1.2 sodium ion channel, which in turn results in impaired neuronal action potential characteristics and weakened signal transmission between neurons, especially in excitatory synaptic transmission. This phenomenon manifests as reduced synaptic plasticity and the formation of “silent synapses.” These changes directly lead to abnormal neural circuits and excitation/inhibition (E/I) imbalance, eventually triggering core autistic traits.

In addition to *SCN2A* being identified as a gene associated with autism and intellectual disability (Sanders, 2015; Ben-Shalom et al., 2017), *SCN9A* has also been reported to be related to susceptibility to autism (Rubinstein et al., 2016). The Nav1.7 voltage-gated sodium channel encoded by the *SCN9A* gene has traditionally been considered to be mainly expressed in the peripheral nervous system. However, recent studies have revealed its important functional mechanism within the central nervous system (Horishita et al., 2017; Kingwell, 2022). Missense mutations in Nav1.7 can lead to channel dysfunction, which in turn significantly reduces the excitability of neurons. This effect is particularly evident in the reduction of the discharge frequency of GABAergic inhibitory neurons. This functional impairment disrupts the delicate balance between excitatory and inhibitory neural transmissions in the brain, constituting a key molecular basis for the onset of autism. Nav1.7 and the key transcriptional regulatory factors in the differentiation process of forebrain neurons have obvious co-expression patterns in time and space. Meanwhile, they show significant enrichment in the hypothalamic region. The hypothalamus is the core brain region that controls social and eating behaviors. These spatial distribution characteristics and functional associations further confirm its potential pathological contribution to the pathogenesis of autism (Rubinstein et al., 2016).

At present, the strategies for treating autism mainly rely on the multidisciplinary intervention model. Early screening, diagnosis, and timely intervention are the key factors to improve the prognosis of patients. With the deepening of our understanding of the pathogenesis, the key role of voltage-gated sodium ion channels (such as Nav1.2 and Nav1.7) and their related molecular networks in the pathophysiology of autism has become increasingly obvious. This indicates that future treatment methods require more precise genetic intervention techniques. Such methods may include conditional gene knock-in techniques for repairing specific mutation sites, or the use of RNA editing techniques to precisely regulate the expression levels of channel proteins. This is more effective than simply completely inhibiting or activating the target gene. Doing so can maximize the therapeutic effect while reducing side effects.

### Voltage-gated sodium channels and functional impairment in the retina

Early studies mainly focused on the mechanisms underlying the role of VGSCs in neurodegenerative diseases (Barbieri et al., 2023; Zhang et al., 2024c). However, many neurodegenerative diseases are often accompanied by retinal dysfunction, and these patients frequently show abnormal expression patterns of VGSCs (Johansson et al., 2019; Cheng et al., 2021). Therefore, the research focus has gradually shifted to clarifying the potential mechanistic roles of these channels in retinal function.

Vision is a complex physiological process. When light passes through the cornea and lens and enters the eye, the retina begins to sense it and convert it into neural signals. This enables people to understand and adapt to the external environment. The retina, as the central organ for the transmission and processing of visual information, largely determines the visual state (Liu et al., 2022). The retina, as a part of the central nervous system, is mainly composed of six types of neurons. These include photoreceptor cells (rods and cones), amacrine cells, bipolar cells, horizontal cells, and ganglion cells (Fu et al., 2023). Among them, photoreceptor cells, which are composed of cone and rod cells, are the main source of visual perception. Cone cells work in bright environments and are responsible for color vision. Rod cells work in dim environments, have high photosensitivity, but lack color discrimination ability (Johansson et al., 2019; Yang et al., 2024). Amacrine cells are located in the inner nuclear layer and mainly regulate synaptic transmission between bipolar cells and ganglion cells by releasing inhibitory neurotransmitters. This facilitates encoding of temporal information and motion detection and is indispensable for the extraction of complex visual features (Sánchez-Sáez et al., 2023; Korympidou et al., 2024). Bipolar cells are key relay neurons that connect photoreceptors and ganglion cells, transmitting signals from photoreceptors to the inner layer of the retina. Functionally, they are divided into ON and OFF types, which respectively respond with depolarization or hyperpolarization to an increase or decrease in light intensity, enhancing the perception of visual contrast (Vielma and Schmachtenberg, 2016; Griffis et al., 2023). Horizontal cells are located in the outer plexiform layer and regulate the electrophysiological activities of adjacent photoreceptors and bipolar cells. These cells enhance visual-contrast and edge-detection capabilities and play an important role in optimizing spatial resolution (Nemitz et al., 2019; Louie et al., 2023). Ganglion cells are the final output neurons of the retina, and their axonal projections form the optic nerve that transmits visual information to brain structures. These neurons exhibit a large number of functional diversifications, including cell subpopulations that selectively respond to specific visual attributes (such as movement, directionality, and color stimuli), thereby performing the final encoding of visual information (Mure et al., 2019; Hu et al., 2022). Photoreceptor cells convert light signals into electrical signals and transmit them to the inner layer of the retina through synapses. In this layer, a series of cells, including bipolar cells, amplify, regulate, and integrate these signals and eventually transmit them to retinal ganglion cells (Zhang et al., 2024b), which then transmit the information to the brain through the optic nerve.

**Figure 5:**
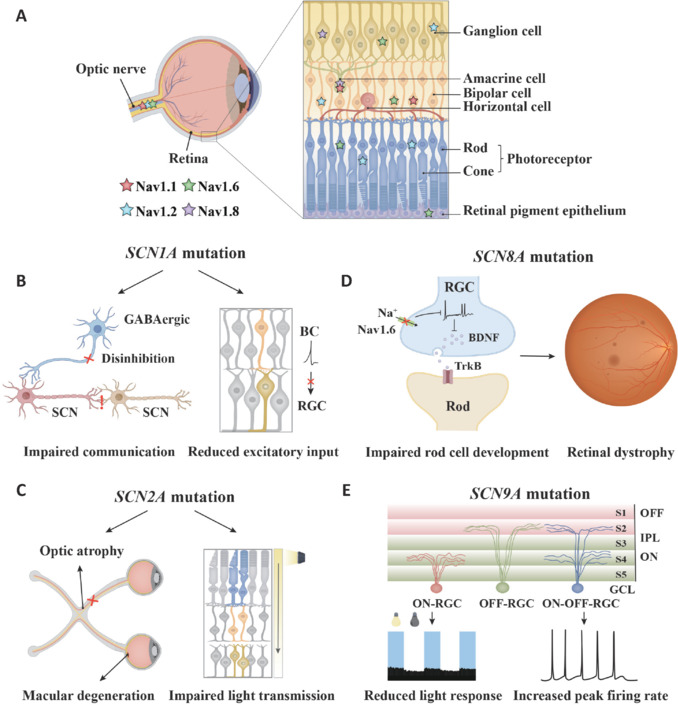
Distribution and function of voltage-gated sodium channels in the retina. (A) Distribution of the four sodium channel subtypes in different retinal cells. (B) *SCN1A* mutation in the retina primarily impairs signal transmission between neurons and reduces excitatory input from the BC to the RGC. (C) *SCN2A* mutation in the retina leads to optic nerve damage and macular degeneration. It also affects photoreceptor (cones and rods) signal transmission and the ability of RGCs to respond to light. (D) *SCN8A* mutation in the retina influences the expression and transport of BDNF, resulting in retinal maldevelopment. (E) *SCN9A* mutation in the retina causes reduced light responses in sustained ON-RGCs and an increased peak firing frequency in transient ON-OFF-RGCs. BC: Bipolar cell; BDNF: brain-derived neurotrophic factor; GCL: ganglion cell layer; IPL: inner plexiform layer; OFF-RGC: OFF-type retinal ganglion cell; ON-OFF-RGCs: ON-OFF retinal ganglion cells; ON-RGC: ON-type retinal ganglion cell; RGC: retinal ganglion cell; SCN: suprachiasmatic nucleus; TrkB: tropomyosin receptor kinase B.

In the complex functional structure of the retina, the precise regulation of neural signals is of vital importance, and VGSCs play a fundamental role in this process. The sodium channel subtypes Nav1.1, Nav1.2, Nav1.6, and Nav1.8 are widely distributed in various retinal cell types (**Figure 5A**). They are involved in the generation and propagation of action potentials. Understanding the complex interactions among these channel subtypes can provide valuable insights to comprehend the functions of retinal circuits and identify potential therapeutic targets for visual impairments.

#### Functions of sodium channel voltage-gated type 1 alpha subunit in the retina

The *SCN1A* gene is highly enriched in key regions of the central nervous system and significantly affects the structure and function of the retina and optic nerve. Nav1.1 is significantly expressed in GABAergic neurons in the initial segments of the axon processes of retinal bipolar neurons, amacrine cells, and the suprachiasmatic nucleus (SCN) (Vahedi et al., 2009; Han et al., 2012; Goaillard et al., 2019). This distribution pattern provides insights into its key role in neural signal transduction, especially in the process of synaptic information transmission.

The retina, as a highly specialized peripheral neurotissue in the central nervous system, is particularly sensitive to gene mutations and disruption of metabolic homeostasis (Xu et al., 2024). Mutations in the gene encoding Nav1.1 can severely disrupt normal retinal function. These mutations alter the electrophysiological characteristics of amacrine cells and bipolar cells, resulting in abnormal retinal electrical signal transmission patterns, which are closely related to dysfunction of the retinal lateral inhibition mechanism. Specifically, the LOF mutation of Nav1.1 may reduce the action potential threshold of amacrine and bipolar cells. This reduction will lead to a decrease in the response threshold of these cells to external stimuli, triggering an abnormal electrical signal cascade response and eventually resulting in transient daily blindness (Vahedi et al., 2009).

In the visual signal transduction pathway, Nav1.1 significantly enhances excitatory synaptic transmission from bipolar cells to retinal ganglion cells by regulating the generation of action potentials. It simultaneously improves the fidelity and efficiency of visual information transmission. This discovery clarifies the functional characteristics of Nav1.1 in bipolar cells and emphasizes its important regulatory role in visual information processing, especially in visual perception functions such as motion detection and temporal resolution (Puthussery et al., 2013).

Furthermore, mutations in the gene encoding Nav1.1 profoundly affect the function of the SCN circuit. As the main circadian pacemaker of mammals, the SCN regulates the physiological rhythms of the system by receiving retinal light input and integrating changes in the daytime environment. Studies have shown that mutations in the gene encoding Nav1.1 reduce transient calcium changes within the SCN and weaken the signal synchronization and coordination among SCN neurons (Han et al., 2012; Sanchez et al., 2019). This leads to a series of circadian rhythm disorders, including but not limited to prolonged circadian cycles and reduced responsiveness to light stimulation (Han et al., 2012; **Figure 5B**). These findings deepen the existing understanding of the central position of the *SCN1A* gene in circadian rhythm regulation and provide important molecular targets for studying the pathogenesis of related diseases and developing potential treatment methods.

#### Functions of sodium channel voltage-gated type 2 alpha subunit in the retina

The Nav1.2 sodium channel shows a highly cell-specific distribution and functional differentiation in the retinal signaling pathway. Nav1.2 shows different expression patterns in photoreceptors, cone bipolar cells, and retinal ganglion cells (RGCs) (Kawai et al., 2005, 2021; Van Wart and Matthews, 2006). In photoreceptors, Nav1.2 provides an ionic basis for photochemical transduction by regulating voltage-dependent membrane depolarization. In cone bipolar cells, Nav1.2-mediated sodium ion influx enhances the depolarization of the presynaptic membrane and promotes the precise release of neurotransmitters. This is crucial for vertical transmission of visual signals.

In RGCs, the expression and function of Nav1.2 show obvious spatiotemporal specificity. During the early developmental stages, Nav1.2 is the main sodium channel subtype in the RGC axons, mainly located in the unmyelinated axon segments and the nascent Ranvier nodes. As myelination occurs, Nav1.2 at these locations is gradually replaced by Nav1.6, although Nav1.2 remains stably expressed in the unmyelinated axonal segments in the retina. This pattern is crucial for maintaining local excitability and signal conduction (Van Wart and Matthews, 2006). Notably, under pathological conditions (such as demyelinating diseases), Nav1.2 expression levels are significantly upregulated, indicating a compensatory regulatory mechanism that may serve as a crucial molecular basis for maintaining visual signal conduction (Craner, 2003).

Electrophysiological studies have further revealed that the rapid sodium ion influx mediated by Nav1.2 plays a unique role in the generation and conduction of RGC action potentials (Kawai et al., 2005, 2021). Selective blocking of Nav1.2 significantly reduced the reactivity of RGCs to light stimulation (**Figure 5C**). This finding indicates that this channel is indispensable in the encoding and transmission of visual information (Risner et al., 2020). This functional specificity is not only obvious under normal physiological conditions, but also particularly prominent when adapting to rapid changes in external light intensity. This provides a molecular basis for rapid adaptation of the visual system.

In conclusion, the Nav1.2 sodium channel forms a complex ion channel network through its differential expression and functional specificity in various retinal neurons. This molecular-level functional differentiation is of great significance in ensuring accurate transmission and processing of visual information. Furthermore, the plasticity changes in this channel under pathological conditions provide potential molecular targets for the treatment of related diseases.

#### Functions of sodium channel voltage-gated type 8 alpha subunit in the retina

Nav1.6 is primarily expressed in the retinal pigment epithelium (RPE), ganglion cells, bipolar cells, and optic nerve cells (Cote et al., 2005; Hossain et al., 2005; Johansson et al., 2019). It plays a crucial role in rapid electrical signal transduction and collaborates with Nav1.8 in the phagocytosis of photoreceptor outer segments (Johansson et al., 2019). Within bipolar cells, Nav1.6 modulates the expression and transport of brain-derived neurotrophic factor (BDNF). Since BDNF and its receptor TrkB are crucial for normal maturation of rod cells and rod pathways, this finding suggests that Nav1.6 may exert essential regulatory effects on rod cell development and function by facilitating the normal functioning of BDNF (Cote et al., 2005; **Figure 5D**).

In RGCs, the functionality of Nav1.6 demonstrates remarkable complexity and criticality. Nav1.6 deficiency in RGCs may promote cell survival by reducing intracellular calcium accumulation (Alrashdi et al., 2019; Werginz et al., 2020a). The low activation threshold and high-frequency firing characteristics of Nav1.6 channels make them irreplaceable in maintaining high-frequency firing and continuously conducting action potentials in RGCs. Nav1.6 shows different expression patterns in dorsal and ventral RGCs. The higher expression levels of Nav1.6 in dorsal RGCs enable them to maintain continuous activity at a higher depolarization level, while ventral cells are more prone to depolarization blockade. This region-specific distribution helps to adapt to the needs of different visual tasks. Because Nav1.6 channels can quickly recover from the inactive state, they can support continuous action potential discharge under high-frequency stimulation, reducing signal loss caused by depolarization blockade (Werginz et al., 2020b).

In the optic nerve, Nav1.6 is particularly concentrated in the Ranvier nodes of myelinated axons, but is not expressed in non-myelinated axons (Boiko et al., 2001). In addition, the function of Nav1.6 also extends to regulating the retinal immune microenvironment. Studies have shown that Nav1.6 not only functions in neurons but also participates in regulating the accumulation and activity of inflammatory cells in the retina, such as macrophages and microglia. These changes in cellular states directly influence the defense and response capabilities of the retina (Alrashdi et al., 2019, 2021).

In conclusion, the Nav1.6 channel performs multiple functions in the retina, including high-frequency discharges in RGCs, regulation of BDNF, region-specific signal processing, and regulation of the immune microenvironment.

#### Functions of sodium channel voltage-gated type 9 alpha subunit in the retina

The Nav1.8 channel is encoded by the *SCN9A* gene and mainly exists in retinal pigment epithelial cells, stellate amacrine cells, and ganglion cells (O’Brien et al., 2008; Johansson et al., 2019). It is resistant to tetrodotoxin, and this characteristic confers it an important role in retinal information processing. In RPE cells, Nav1.8 helps phagocytize the outer segments of photoreceptors. It accumulates in the phagocytic body and interacts directly with it, which is crucial for maintaining retinal health (Johansson et al., 2019). In stellate amacrine cells, the Nav1.8 channel maintains the oscillatory activity of the cells. This oscillation is an important component of the retina’s response to light stimulation, especially to flashes. Researchers found that blocking Nav1.8 significantly weakened the oscillatory potential of stellate amacrine cells. Thus, Nav1.8 is crucial for maintaining the synchronization of electrical activity and conduction efficiency of these cells.

In RGCs, the Nav1.8 current acts on the cell body and proximal dendrites. It may directly participate in the formation of dendritic action potentials, thereby regulating information integration and signal transmission. Notably, Nav1.8 is particularly expressed in large-diameter RGC axons but not in unmyelinated axons, indicating that it plays a specific role in specific RGC types (O’Brien et al., 2008). In addition, Nav1.8 is deeply involved in the functional adjustment of specific RGC types. Its influence range is very wide, ranging from weakening the light response of sustained ON cells to regulating the firing pattern of transient ON-OFF cells through inhibitory pathways (**Figure 5E**). RGCs can be classified into three types: ON-type RGCs, which are excited by an increase of light intensity, OFF-type RGCs that are excited by the decrease in light intensity, and ON-OFF type RGCs that respond to changes in light intensity. Specifically, blocking Nav1.8 will significantly reduce the response of sustained ON cells to light stimulation, while the peak firing frequency of transient ON-OFF cells will increase. This discovery indicates the diverse functions of Nav1.8 in different types of RGCs, which is crucial for maintaining the complex and precise signal processing network within the retina (Smith et al., 2017).

The retina contains four major voltage-gated sodium channel subtypes—Nav1.1, Nav1.2, Nav1.6, and Nav1.8—and their expression patterns and functions vary in different retinal cells. The Nav1.1 channel mainly regulates the electrophysiological characteristics of bipolar cells and amacrine cells and is also involved in the regulation of circadian rhythms. Nav1.2 channels play a key role in photoreceptors and RGCs and show obvious spatiotemporal specificity during early development and myelin formation. Nav1.6 channels regulate the expression and transport of BDNF and help neurons maintain high-frequency discharges, and can also regulate the immune microenvironment, affecting the overall homeostasis of the retina. Nav1.8 channels show unique anti-tetrodotoxin properties. Research has shown that these channels are important for the phagocytic activity of RPE and also participate in the signal processing of different types of RGCs.

These channels jointly form a complex network, ensuring accurate transmission and processing of visual information while providing potential therapeutic targets for related diseases. An in-depth understanding of the functional mechanisms of these sodium channels can help clarify the molecular basis of visual information processing and open up new directions for the diagnosis and treatment of retinal-related diseases. The core role of these ion channels in maintaining normal visual function deserves further exploration.

## Voltage-Gated Sodium Channels and Neuronal Regeneration

The voltage-gated sodium channel is one of the most fundamental ion conduction pathways in the nervous system. It plays a key role in maintaining neuronal homeostasis and coordinating neural repair mechanisms.

Many studies have shown that damage to neurons can lead to significant changes in the expression patterns and functional characteristics of these channels (Brown et al., 2023; Wang et al., 2024). These adaptive changes have dual effects, acutely regulating excitotoxicity and the cell death cascade while chronically mediating key repair processes, including axon regeneration, synaptic plasticity, and functional recovery.

### Dynamic expression and functional regulation of voltage-gated sodium channels in neuronal differentiation

VGSCs exhibit precise time-regulated characteristics during the process of neuronal differentiation. There is a significant association between the expression pattern of sodium channels and the acquisition of neuronal function. This development process is divided into three key stages. In the early stage of neuronal differentiation, neurons start to express specific markers, but their functions are not yet mature. At this point, the expression of sodium channels remains relatively low, which is only sufficient to generate immature action potentials. During the intermediate stage of development, as neurons establish synaptic connections, the expression of sodium channels is significantly upregulated. This enables neurons to generate a single mature action potential. In the late differentiation stage, neurons reach functional maturity and establish an integrated neural network. Moreover, the amplitude of the sodium current (INa) tends to stabilize. Through synergistic effects with other ion channels, especially type IA potassium channels, neurons acquire the ability to repeatedly generate action potentials (Song et al., 2013).

During this complex developmental process, different sodium channel subtypes exhibit unique spatiotemporal expression patterns and functional characteristics. Nav1.2 is a major contributor of the sodium current to central neurons during the embryonic and early developmental stages. It plays a fundamental role in the generation of action potentials, retrograde propagation, and synaptic integration. Specifically, during the early differentiation of neurons, Nav1.2 is located mainly in the cell body and dendrite regions. With the progression of differentiation, its expression area expands to the initial segment of the axonal process and forms characteristic clusters in the axonal conduction area. Notably, when Nav1.2 reaches maturity, its expression is gradually replaced by Nav1.6. This transformation is crucial for neuronal maturation and the establishment of synaptic plasticity (Brown et al., 2023).

The expression level of Nav1.6 is relatively low during early neuronal development, but it is significantly upregulated during neuronal differentiation and maturation. In mature neurons, Nav1.6 becomes the main channel responsible for generating neuronal sodium currents in the central nervous system. It maintains normal neuronal excitability by precisely regulating persistent and recurrent sodium currents and plays a key role in action potential repolarization at the same time. Nav1.6 is mainly localized in the AIS and nodes of Ranvier, and this specific localization depends on its molecular interaction with ankyrin-G (Tidball et al., 2020).

Unlike Nav1.2 and Nav1.6, Nav1.7 exhibits unique developmental dynamics in sensory neurons. In early differentiation, the expression of Nav1.7 is relatively low and is mainly confined to the endoplasmic reticulum region surrounding the cell body. With the progression of differentiation, its expression is significantly upregulated and extends to the cell membrane surface, especially at the ends of axons and the nodes of Ranvier of myelin axons. The main function of Nav1.7 is to regulate neuronal excitability, especially by amplifying subthreshold depolarization stimulation through its unique slow inactivation kinetics, thereby promoting the generation of action potentials (McDermott et al., 2019).

The regulatory mechanism of VGSCs in neuronal differentiation is very precise and involves the synergistic effect of multiple regulatory factors. These factors include BDNF, nerve growth factor, vascular endothelial growth factor, and glial cell-derived neurotrophic factor (Hingorani et al., 2024). Among these factors, BDNF has a significant effect on the expression and function of sodium channels through a unique two-phase regulatory mechanism. Specifically, when 40 ng/mL BDNF was added during the differentiation of neural stem cells, in the early differentiation stage, BDNF doubled the sodium current density compared with that in the control group. Although this enhancement is not sufficient to induce an action potential, it marks the transformation of cells from a proliferative state to a differentiated state. In the late differentiation stage, BDNF accelerates the inactivation of sodium channels. This mechanism reduces the sodium current density to one-third of that of the control group through the TrkB, p75, and Fyn signaling pathways. This precise two-phase regulation ensures the appropriate maintenance of excitability during the process of neuronal differentiation (Leng et al., 2009). In addition to biochemical regulators, physical factors such as mechanical stress also play crucial roles in the regulation of sodium channel function. Research has shown that the epithelial sodium channel (ENaC) can act as a mechanosensor to detect cerebrospinal fluid shear forces, triggering downstream signaling cascades by mediating sodium ion influx. ENaC-mediated mechanotransduction participates in regulating neural stem cell proliferation and differentiation by modulating the opening of calcium release-activated channels and ERK kinase phosphorylation levels. Notably, ENaC functional deficiency not only affects the proliferative capacity of neural stem cells and neuroblasts but also leads to a significant reduction in newborn neurons (Petrik et al., 2018). Under pathological conditions, VGSCs also play complex and crucial regulatory roles in tumor stem cell differentiation. Recent studies have revealed that VGSCs maintain glioblastoma (GBM) stem cell properties by precisely regulating membrane potential homeostasis, and their elevated expression significantly correlates with poor prognosis in proneural GBM patients (Ai et al., 2023; Giammello et al., 2024). In-depth mechanistic exploration revealed that VGSCs can regulate the expression of the key transcription factors SOX2 and NANOG in GBM stem cells by negatively regulating the ERK1/2 and AKT signaling pathways, affecting cell cycle dynamics. When specific sodium channel blockers, such as the tetrodotoxin TTX or riluzole, are used for intervention, they can induce cells in the G0 and G2/M phases to transition to the G1 phase while significantly reducing GBM stem cell characteristics and self-renewal ability. Notably, this VGSC-mediated regulatory mechanism is closely related to the resistance of GBM to temozolomide. The combined application of VGSC inhibitors and the ERK1/2 pathway activator RB5 can significantly increase the antitumor efficacy of temozolomide (Giammello et al., 2024). Overall, VGSCs primarily promote GBM progression through two key aspects: maintaining tumor stem cell properties and regulating chemotherapy resistance. Therefore, targeting VGSC activity not only provides a new perspective for understanding GBM pathogenesis but also offers important potential targets for developing innovative therapeutic strategies.

In summary, VGSCs regulate neuronal differentiation through complex and precise mechanisms. Different VGSC subtypes work collaboratively to control neuronal development. They achieve this through specific expression patterns that vary depending on location and time. Their functional characteristics also play a key role. This regulation involves not only the coordinated effect of multiple molecular signaling pathways but also physical factors such as mechanical stress. These findings help us better understand the basic mechanisms of neural development. These findings also provide valuable insights for the development of treatment methods for related diseases.

### Functional role of voltage-gated sodium channels in neuronal regeneration

Injury to VGSCs can cause cascading changes in the functions of multiple key neuronal processes. In dendrites, damaged sodium channels can reduce the depolarization ability of local dendrites. This limits the inward flow of calcium ions into dendritic spines, disrupts the backpropagation of action potentials, and weakens calcium signal transduction. These effects impair synaptic plasticity and affect the information integration ability of dendrites (Spratt et al., 2019). In axons, dysfunction of sodium channels directly interferes with the generation and conduction of action potentials. This slows the conduction speed, reduces the magnitude of the action potential, and sometimes even completely blocks the signal. It also damages axonal transport and affects the production and release of neurotransmitters (Bunton-Stasyshyn et al., 2019). At the synapse, abnormal action potentials caused by sodium channel dysfunction reduce the amount of calcium entering the presynaptic terminal. This reduces the release of neurotransmitters and weakens synaptic transmission. Specifically, excitatory transmission becomes less effective, and the balance of AMPA/NMDA receptors changes. These synaptic problems further affect the formation and maintenance of synaptic plasticity (Brown et al., 2023). From the perspective of neural networks, the functional impairment of these individual neurons, coupled with alterations in the excitatory-inhibitory balance, reduces the information encoding, transmission and integration capabilities of neural circuits.

Researchers have begun to explore therapeutic strategies that utilize VGSC regulation to promote neuronal regeneration and functional recovery. Xue et al. (2014) showed that the expression of prokaryotic VGSCs in specific neurons through genetic engineering can precisely control the excitability of the target neuron population. This provides a key tool for studying activity-dependent synaptic plasticity. Recently, the Hingorani research team has made breakthroughs in the treatment of spinal cord injury (SCI) (Hingorani et al., 2024). They developed an innovative cell therapy strategy. This strategy involves transplanting genetically engineered dorsal root ganglion neurons overexpressing bacterial VGSC (NaChBac) into a mouse model of SCI. The results show that NaChBac promotes functional recovery after SCI through multiple synergistic mechanisms. First, the expression of NaChBac reduces the neuronal activation threshold and enhances intrinsic excitability. This increased the intracellular levels of calcium ions and cAMP and subsequently activated the AKT/mTOR survival signaling pathway. This process increases the expression of the antiapoptotic protein Bcl2 and simultaneously inhibits the expression of the proapoptotic protein Caspase-3, thereby increasing the survival ability of neurons. Second, increased neuronal activity induces increased secretion of various neurotrophic factors, including BDNF, glial cell-derived neurotrophic factor and nerve growth factor. This creates a microenvironment conducive to the survival of neurons and the growth of axons. In addition, the expression of NaChBac enhances the migration ability of transplanted neurons and promotes the preservation of nerve fibers and myelin formation. It also regulates the synaptic recombination pattern, increasing excitatory synaptic input (VGLUT2-positive contact) while reducing inhibitory input (VGAT-positive contact). These synergistic mechanisms eventually establish new relay neural circuits at the injury site, maintaining the excitatory activity of neurons in the distal injury area. Notably, this treatment strategy significantly improved the recovery of motor function without affecting pain sensitivity. These findings provide a new potential direction for the clinical treatment of SCI (Hingorani et al., 2024).

**Figure 6:**
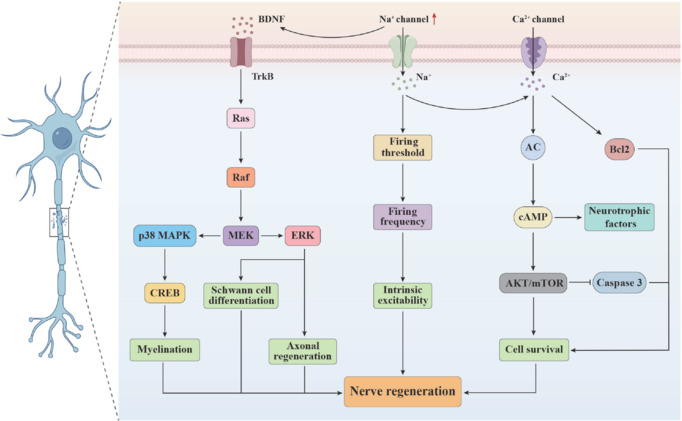
Molecular mechanisms of neuronal regeneration mediated by VGSCs. VGSC promotes neuronal regeneration through three key signaling pathways: (1) activation of the BDNF/TrkB-Ras-ERK-MAPK cascade to facilitate myelination and axonal regeneration; (2) regulation of sodium ion influx to increase neuronal excitability; and (3) modulation of the calcium channel-cAMP-neurotrophic factor-AKT/mTOR axis to promote neuronal survival. AC: Adenylate cyclase; AKT/mTOR: protein kinase B/mammalian target of rapamycin; Bcl2: B-cell lymphoma 2; BDNF: brain-derived neurotrophic factor; cAMP: cyclic adenosine monophosphate; CREB: cAMP response element-binding protein; ERK: extracellular signal-regulated kinase; MEK: MAPK/ERK kinase; p38 MAPK: p38 mitogen-activated protein kinase; Raf: rapidly accelerated fibrosarcoma; Ras: rat sarcoma; TrkB: tropomyosin receptor kinase B; VGSC: voltage-gated sodium channel.

In conclusion, VGSCs, as crucial ion channels in the nervous system, promote neuronal regeneration through three primary molecular mechanisms: (1) activation of the BDNF/TrkB-Ras-ERK-MAPK signaling pathway, which promotes myelin formation and axonal regeneration; (2) regulation of sodium ion influx, which enhances neuronal excitability; and (3) activation of the calcium channel-cAMP-neurotrophic factor-AKT/mTOR signaling axis, which promotes cellular survival (**Figure 6**). The synergistic effects of these mechanisms are not only essential for maintaining normal neuronal function but also play a central role in postinjury repair processes. In particular, VGSCs function as “molecular switches” in neuronal regeneration by regulating neuronal excitability, axonal growth, synaptic reconstruction, and neurotrophic factor expression. Therefore, a comprehensive understanding of VGSC-mediated neuronal regeneration mechanisms will provide crucial theoretical foundations for developing targeted therapeutic strategies for neurological injuries, pioneering new pathways for treatment.

### The sodium channel and the renin-angiotensin system

The renin‒angiotensin system plays an important role in maintaining cardiovascular homeostasis, regulating fluid balance, and controlling electrolyte metabolism. The entire process begins with angiotensinogen synthesis by the liver. When the kidneys sense signals such as a drop in blood pressure or a reduction in blood volume, juxtaglomerular cells release renin. Renin, as a protease, can cleave angiotensinogen into angiotensin I. In addition, angiotensin-converting enzyme, which mainly exists in the vascular endothelium of the lungs and kidneys, converts angiotensin I into the active substance angiotensin II (AngII). AngII is the core effector molecule of this system. After binding to the AngII type 1 receptor (AT1 receptor), it causes vasoconstriction and stimulates the adrenal cortex to release aldosterone (Martyniak and Tomasik, 2022).

AngII affects sodium reabsorption through two main pathways. It can directly activate the ENaC in the collecting duct through the AT1 receptor, and this process is not dependent on aldosterone. Moreover, it also promotes the release of aldosterone by the adrenal glands. When aldosterone binds to the mineralocorticoid receptor in the distal nephron, it enhances the activity of ENaC and the function of the NaCl cotransporter in the distal convoluted tubules. These effects jointly promote sodium retention and potassium excretion (Sealey and Blumenfeld, 2025). The prorenin receptor in the collecting duct also plays an important role in this network. It not only promotes the formation of local AngII but also directly enhances the activity of ENaC through the PKA–AKT signaling pathway (Prieto et al., 2021).

This system has a self-regulating mechanism. When dense spot cells sense the reduction in sodium reaching the distal tubules, they stimulate renin secretion, forming a negative feedback loop. When sodium channel function is abnormal or RAS regulation is imbalanced, excessive or insufficient sodium retention may occur, leading to clinical problems such as hypertension, edema or electrolyte imbalance (Kim et al., 2012; Wu et al., 2018). Therefore, these sodium channels are not only important components of the RAS but also the preferred targets for the treatment of related diseases.

## Voltage-Gated Sodium Channels and Therapeutic Strategies

**Figure 7:**
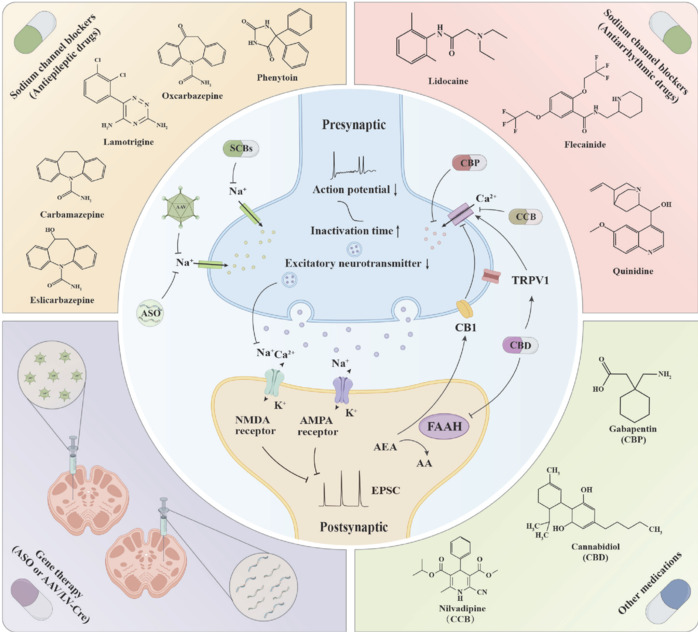
Drugs and mechanisms for treating sodium channel dysfunction. The diagram illustrates four classes of drugs used to treat diseases caused by sodium channel dysfunction, including antiepileptic drugs (top left), which primarily target sodium ion channels; antiarrhythmic drugs (top right), which regulate cardiac sodium channels; gene therapy (bottom left), which restores sodium channel function through gene repair or modulation; and antiepileptic drugs that act on other receptors (bottom right), which indirectly modulate sodium channel function by affecting different receptors. The center of the diagram displays the mechanisms of action for these four drug classes. AA: Arachidonic acid; AEA: anandamide; AMPA: α-amino-3-hydroxy-5-methyl-4-isoxazolepropionic acid; CB1: cannabinoid receptor 1; CBP: gabapentin; CCB: calcium channel blocker; EPSC: excitatory postsynaptic current; FAAH: fatty acid amide hydrolase; NMDA: N-methyl-D-aspartate; SCBs: sodium channel blockers; TRPV1: transient receptor potential vanilloid 1.

Dysfunction of VGSCs plays a key role in the pathogenesis of various neurological disorders, including epilepsy, migraine, and ASD (**Additional Table 2**). Current therapeutic strategies for VGSC-related diseases can be classified into two main categories: traditional pharmacological interventions and emerging gene therapies (**Figure 7**).

**Additional Table 2:** Summary of voltage-gated sodium channel gene mutations and their associated pathological manifestations in neurological disorders

Gene	Mutation type	Disease	Pathology description	Reference
*SCN1A*	Nav1.1 GOF	Familial hemiplegic migraine type 3	Severe migraines with neurological symptoms, triggered by sensory stimuli such as light and noise.	Shao et al., 2018
	Nav1.1 LOF	Dravet syndrome	Severe, frequent seizures starting in infancy with fever sensitivity, leading to developmental delays and cognitive impairment.	Myers, 2023
	Nav1.1 LOF	Generalized epilepsy with febrile seizures plus	Multiple types of seizures beginning with febrile episodes in childhood, persisting beyond typical age with various seizure patterns.	Dimova et al., 2010
	Nav1.1 LOF	Autism spectrum disorder	Persistent social interaction and communication difficulties with restricted interests and repetitive behaviors, typically emerging in early childhood.	Papp-Hertelendi et al., 2018
	Nav1.1 LOF	Epileptic encephalopathy	Severe, uncontrolled seizures causing progressive cognitive decline and developmental regression, often with behavioral changes.	Clatot et al., 2023
*SCN2A*	Nav1.2 GOF	Epileptic encephalopathy	Intractable seizures with cognitive, language, and behavioral developmental disorders.	Jia et al., 2024
	Nav1.2 GOF	Early-onset epilepsy	Seizures starting shortly after birth or infancy, often with developmental delays and neurological problems.	Adney et al., 2020
	Nav1.2 GOF	Migraine	Recurrent unilateral headaches with nausea, vomiting, visual disturbances, and light or sound sensitivity.	Kowalska et al., 2020
	Nav1.2 LOF	Autism spectrum disorder	Social interaction and communication difficulties, along with stereotypical and repetitive behaviors.	Ben-Shalom et al., 2017
*SCN3A*	Nav1.3 GOF	Early-onset epilepsy	Seizures starting shortly after birth, possibly with developmental delays and neurological abnormalities.	Thuresson et al., 2017
	Nav1.3 GOF	Parkinson's disease	Degeneration of substantia nigra dopaminergic neurons and Lewy body formation leading to tremor, rigidity, bradykinesia, and postural instability.	Saleh et al., 2024
*SCN4A*	Nav1.4 GOF	Hyperkalemic periodic paralysis	Periodic muscle weakness or paralysis triggered by high potassium levels, often accompanied by muscle stiffness.	Weber, 1993
	Nav1.4 LOF	Hypokalemic periodic paralysis type 2	Periodic muscle weakness, usually with low blood potassium levels, worsening after rest or exposure to coldness.	Jurkat-Rott et al., 2000
	Nav1.4 LOF	Congenital myasthenic syndrome	Muscle weakness and fatigability from birth, characterized by impaired neuromuscular transmission, affecting voluntary muscle function and motor development.	Estephan et al., 2022
*SCN5A*	Nav1.5 GOF	Long QT syndrome type 3	Characterized by prolonged QT intervals leading to life-threatening arrhythmias, particularly torsades de pointes during rest or sleep	Cano et al., 2020
	Nav1.5 LOF	Brugada syndrome	Typical presentation includes right bundle branch block, ST-segment elevation in precordial leads, with high risk of ventricular arrhythmias or sudden cardiac death.	Walsh et al., 2025
	Nav1.5 LOF	Sudden infant death syndrome	Sudden unexplained death in infants during sleep, usually within 6 months after birth.	Tester et al., 2018
	Nav1.5 LOF	Progressive cardiac conduction disease	Gradual degeneration of the heart conduction system, leading to sinus node failure and conduction block, potentially causing heart failure and sudden death.	Derangeon et al., 2017
*SCN8A*	Nav1.6 GOF	Epileptic encephalopathy	Severe, uncontrolled seizures accompanied by progressive cognitive decline and developmental regression.	Gardella et al., 2018
	Nav1.6 LOF	Autism spectrum disorder	Social interaction and communication difficulties, along with stereotypical, repetitive behaviors.	Mahjani et al., 2021
	Nav1.6 LOF	Intellectual Disability	Significant limitations in cognitive functioning and adaptive skills affecting daily life, manifesting before the age of 18 years.	Liu et al., 2019
*SCN9A*	Nav1.7 LOF	Congenital insensitivity to pain	Complete or partial loss of pain sensation, leading to an inability to perceive bodily pain.	Yammine et al., 2023
*SCN10A*	Nav1.8 GOF	Painful neuropathy	Chronic pain with burning, stabbing, or shock-like sensations, hypersensitivity to mild stimuli, hyperalgesia and allodynia.	Huang et al., 2014
	Nav1.8 LOF	Congenital insensitivity to pain	Complete or partial loss of pain sensation, leading to an inability to perceive bodily pain.	Palma et al., 2021
*SCN11A*	Nav1.9 GOF	Painful neuropathy	Recurrent, unexplained pain, difficult to diagnose and treat, with hard-to-identify symptoms.	Hoeijmakers et al., 2015
	Nav1.9 LOF	Congenital insensitivity to pain	Complete or partial loss of pain sensation, leading to an inability to perceive bodily pain.	Palma et al., 2021

This table systematically summarizes the associations between voltage-gated sodium channel gene mutations and neurological disorders, detailing the types of mutations in different genes (gain-of-function/loss-of-function) and their related diseases with pathological manifestations. This comprehensive summary has significant clinical application value, not only helping physicians better understand the pathophysiological mechanisms of voltage-gated sodium channel gene mutations in neurological disorders and providing more precise diagnostic criteria for patients but also offering a crucial theoretical foundation for developing individualized treatment strategies targeting specific gene mutations. GOF: Gain-of-function; LOF: loss-of-function.

Among conventional drugs, sodium channel blockers are first-line medications for the treatment of various ion channel diseases. This type of drug can be divided into two main categories: antiepileptic drugs and antiarrhythmic drugs. Antiepileptic sodium channel blockers include carbamazepine, eslicarbazepine, lamotrigine, oxcarbazepine, and phenytoin (Kong et al., 2015; Braakman et al., 2017; Asadi-Pooya et al., 2023). These drugs effectively control epileptic seizures caused by GOF mutations by blocking sodium channels and reducing excessive neuronal discharge. In addition to their use in treating epilepsy, they are also used to treat other diseases related to sodium ion channels. Lamotrigine can be used to treat myotonia. The lamotrigine derivatives developed by Pfizer can be used as selective blockers of Nav1.8 to treat pain. Oxcarbazepine is often used for pain control in trigeminal neuralgia and certain hereditary neuropathies (Alsaloum et al., 2025).

The main antiarrhythmic sodium channel blockers are lidocaine, flecainide, and quinidine (Fukuda et al., 2011; Kong et al., 2015). These drugs have a high affinity for the Nav1.5 sodium channel in the heart and are often used to treat heart diseases such as LQT3 long QT syndrome and Brugada syndrome. Lidocaine works by enhancing the rapid inactivation of sodium channels. It can be used not only for arrhythmia but also as a local anesthetic and a symptom relief drug for myotonia. Flecainide can effectively block the open state of sodium channels and is particularly suitable for correcting the abnormal inactivation of Nav1.5 variants. Quinidine can reduce the peak and late sodium currents simultaneously and is the main therapeutic drug for Brugada syndrome (Alsaloum et al., 2025). However, traditional sodium channel blockers have limitations. Owing to their lack of selectivity, they may simultaneously inhibit the functions of normal and pathological sodium channels. Take the DS as an example. This disease is caused mainly by the loss of Nav1.1 function, which leads to a reduction in GABAergic interneuron activity. Nonselective sodium channel blockers may further inhibit residual Nav1.1 function, instead aggravating symptoms (Dalic et al., 2015; Snoeijen-Schouwenaars et al., 2015; Zographos et al., 2022; Myers, 2023).

In addition to traditional sodium channel blockers, drugs with other mechanisms of action have also shown therapeutic potential. Gabapenti, a GABA analog, reduces the release of excitatory neurotransmitters by binding to the α2δ subunit of calcium channels (Panebianco et al., 2021; Russo et al., 2023; Escobar-Espinal et al., 2024). Cannabidiol regulates neuronal excitability through the TRPV1 receptor and the endogenous cannabinoid system (Manzoni et al., 2025). Calcium channel blockers can also effectively regulate neuronal activity by inhibiting calcium influx (Ghosh et al., 2023). These drugs jointly regulate neuronal excitability from different perspectives and provide symptom relief (**Figure 7**).

In recent years, gene therapy has shown great potential in the treatment of ion channel diseases. DS is a typical case in this field. The STK-001 drug effectively alleviates epileptic symptoms by inhibiting abnormal splicing events (such as the insertion of exon 20 N) and increasing the levels of functional *SCN1A* mRNA and Nav1.1 protein (Pong et al., 2022; Wengert et al., 2022). This drug has successfully completed a phase I/II clinical trial (NCT04442295) and is currently undergoing a phase II study (NCT04740476). Clinical data show that the frequency of epileptic seizures in patients has significantly decreased, and their cognitive and behavioral functions have also improved, which provides new treatment hope for patients with DS. Another innovative therapy, ETX101, uses the AAV9 vector to deliver specific transcription factors to increase endogenous SCN1A expression (Perucca et al., 2023; Bialer et al., 2024). The safety and efficacy of encoded therapeutics have been evaluated in patients aged 2–30 years in three phase I/II clinical trials (NCT05419492). Two studies have confirmed that even in the advanced stage of the disease, restoring Nav1.1 expression can significantly improve clinical symptoms (Tanenhaus et al., 2022; Gao et al., 2023).

The innovative pipeline in the field of epilepsy treatment is both rich and diverse. PRAX-222 has been granted orphan drug designation by the FDA for *SCN2A* gain-of-function epilepsy (Alsaloum et al., 2025). This therapy utilizes an AAV vector to deliver antisense RNA, specifically targeting the overexpression of *SCN2A*. Praxis has obtained patent protection for this technology and received a $2 million grant from the NIH Rare Disease Research Network. Additionally, NBI-921352, a selective Nav1.6 inhibitor, is currently being evaluated for its therapeutic effects on *SCN8A*-related epilepsy in a phase II clinical trial (NCT04873869) (Bialer et al., 2022; Johnson et al., 2022). This project is led by Neurocrine Biosciences and is supported by the National Institutes of Health (NIH) Institute of Neurological Disorders and Stroke. Furthermore, Prax562 specifically inhibits the persistent sodium current (INaP) of Nav1.6, rather than the transient sodium current (INaT), and has entered phase II clinical trials (NCT05818553) (Kahlig et al., 2022; Müller et al., 2024). This unique mechanism is expected to reduce the adverse reactions commonly associated with traditional antiepileptic drugs.

A key breakthrough has been made in the field of pain treatment. In 2023, VX-548 (Suzetrigine), a selective inhibitor of Nav1.8 developed by Vertex, was approved by the FDA for the treatment of moderate to severe acute pain (Jones et al., 2023; Vaelli et al., 2024). This is the first marketed drug targeting Nav1.8. The company is evaluating its efficacy in treating painful diabetic peripheral neuropathy in a phase III clinical study (NCT06628908). SiteOne Therapeutics’ ST-2427 is a Nav1.7 inhibitor modified on the basis of a natural toxin and is currently in the phase I clinical trial (NCT04475198) stage. Two Nav1.8 inhibitors from Latigo, LTGO-305 and LTGO-001, are also under phase I clinical evaluation (NCT06554574) (Alsaloum et al., 2025).

Substantial progress has been made in drug repositioning strategies in this field. Studies have confirmed that existing drugs such as carvedilol, amitriptyline, and nifedipine can effectively inhibit mutant sodium channels (Atkin et al., 2018; Wong and Escayg, 2024). Notably, the application of lacosamide in patients with small-fiber neuropathy, especially those with marked effects on those carrying specific Nav1.7 variations, is particularly important (Alsaloum et al., 2025).

With the deepening of the concept of precision medicine and the maturation of gene editing technology, personalized treatment strategies that integrate the advantages of drugs and gene therapy will emerge in the future. The FDA has established the regenerative medicine advanced therapy (RMAT) designation to accelerate the review process of innovative therapies for rare diseases related to sodium channels. These measures will provide patients with more precise and effective treatment plans, significantly improving disease prognosis and quality of life.

## Limitations

This review has several limitations that need to be noted. First, the VGSC family has a wide variety of member types, each with distinct expression patterns in different neurons, leading to varying effects on diseases (Alsaloum et al., 2025). In this review, we focused on several VGSC subtypes with relatively high expression levels in central neurons, emphasizing their mechanism of action and functional characteristics in neurodegenerative diseases. However, we did not comprehensively describe the roles of all sodium channel subtypes in various neurological diseases. In particular, the regulatory effects of Nav1.7, Nav1.8, and Nav1.9 in the peripheral nervous system on neuropathic pain have not been thoroughly analyzed. Second, we have summarized the traditional drugs and novel gene therapies for treating sodium channel-related diseases. However, the description of key clinical information, such as the effective dose range of drugs, dose-dependent effects, long-term medication safety, and potential toxicity and side effects, is still insufficient. These contents, which are crucial for guiding clinical medication, need to be further supplemented and improved in future research. Third, although progress has been made in the research of VGSCs as “molecular switches” for neural regeneration in recent years, strong evidence from human clinical studies is still lacking. These findings indicate that our understanding of how sodium channels regulate nerve regeneration and their application value in treatment remains an area that requires further exploration. There are still many problems and challenges to be addressed in the transformation process from basic research to clinical application.

## Conclusions

Many studies have shown that VGSCs exhibit functional abnormalities in various neurological diseases. In research on migraines, epilepsy, autism, and retinal diseases, scientists have tested various regulatory approaches targeting specific sodium channel subtypes in cell and animal models, including small-molecule drugs, gene therapy, and antisense oligonucleotides, all of which have shown promising results in preclinical studies.

However, one major challenge in current research is determining the precise roles of different sodium channel subtypes in specific disease processes. For example, loss of Nav1.1 channel function can cause epilepsy, but not all Nav1.1 functional impairments lead to the same clinical symptoms. Neurological diseases are inherently complex. Although we have established many disease models, animal models cannot fully replicate all the characteristics of human diseases. Therefore, an important direction for future research is developing tools and detection methods that can precisely identify specific sodium channel subtypes. This approach will help us better understand the dynamic changes in various sodium channels during disease progression and deepen our understanding of their specific mechanisms in different neurological diseases.

Our review makes new contributions in several aspects. First, we systematically analyzed how sodium channel dysfunction contributes to migraines, epilepsy, autism, and retinal diseases, particularly revealing the complex mechanisms by which sodium channels participate in visual signal processing. These findings provide a theoretical foundation for the precise diagnosis and targeted treatment of related diseases. Second, we detail the three core mechanisms by which sodium channels promote neuronal regeneration, offering new perspectives for developing neural repair therapies. Third, we reviewed the evolution of treatment technologies for sodium channel-related diseases, from traditional drugs to novel specific modulators to gene therapy, and evaluated innovative therapies currently in preclinical and clinical trial stages, providing valuable references for future research.

Although therapeutic approaches targeting sodium channels show promising prospects in various disease models, several key issues must be addressed in the transition from laboratory to clinical application. First, we need to confirm whether new sodium channel modulators can effectively cross the blood‒brain barrier and evaluate their distribution and activity in the central nervous system. Second, current cell models cannot fully reflect complex neural networks and intercellular interactions in real disease environments, so the efficacy of these drugs needs verification under conditions closer to physiological settings. Additionally, we need to comprehensively assess the effects of long-term modulation of specific sodium channel subtypes on neuronal function, synaptic plasticity, and neural network reorganization. Notably, gene therapy approaches such as STK-001 (for DS) and ETX101 (for enhancing *SCN1A* gene expression) have shown encouraging results and represent cutting-edge directions in the treatment of sodium channel-related diseases. However, the precision, durability, and safety of gene delivery systems, as well as methods for precisely controlling gene expression levels, still require further investigation in more complex animal models.

## Additional files:

***Additional Table 1:*** *Molecular and pharmacological characteristics of voltage-gated sodium channel subtypes.*

***Additional Table 2:*** *Summary of voltage-gated sodium channel gene mutations and their associated pathological manifestations in neurological disorders.*

## Data Availability

*All relevant data are within the paper and its Additional files*.
